# Machine Learning Tailored Anodes for Efficient Hydrogen Energy Generation in Proton-Conducting Solid Oxide Electrolysis Cells

**DOI:** 10.1007/s40820-025-01764-7

**Published:** 2025-05-23

**Authors:** Fangyuan Zheng, Baoyin Yuan, Youfeng Cai, Huanxin Xiang, Chunmei Tang, Ling Meng, Lei Du, Xiting Zhang, Feng Jiao, Yoshitaka Aoki, Ning Wang, Siyu Ye

**Affiliations:** 1 Huangpu Hydrogen Energy Innovation Center, School of Chemistry and Chemical Engineering, Guangzhou University, Guangzhou, 510006 People’s Republic of China; 2 School of Mathematics and Information Science, Guangzhou University, Guangzhou, 510006 People’s Republic of China; 3 School of Materials Science and Engineering, Xihua University, Chengdu, 610039 People’s Republic of China; 4 Faculty of Engineering, Hokkaido University, N13W8, Kita-Ku, Sapporo, 060-8628 Japan

**Keywords:** Machine learning, Proton-conducting solid oxide electrolysis cells, Hydrogen energy, Anode

## Abstract

In the global trend of vigorously developing hydrogen energy, proton-conducting solid oxide electrolysis cells (P-SOECs) have attracted significant attention due to their advantages of high efficiency and not requiring precious metals. However, the application of P-SOECs faces challenges, particularly in developing high-performance anodes possessing both high catalytic activity and ionic conductivity. In this study, La_0.9_Ba_0.1_Co_0.7_Ni_0.3_O_3−*δ*_ (LBCN9173) and La_0.9_Ca_0.1_Co_0.7_Ni_0.3_O_3−*δ*_ (LCCN9173) oxides are tailored as promising anodes by machine learning model, achieving the synergistic enhancement of water oxidation reaction kinetics and proton conduction, which is confirmed by comprehensively analyzing experiment and density functional theory calculation results. Furthermore, the anodic reaction mechanisms for P-SOECs with these anodes are elucidated by analyzing distribution of relaxation time spectra and Gibbs energy of water oxidation reaction, manifesting that the dissociation of H_2_O is facilitated on LBCN9173 anode. As a result, P-SOEC with LBCN9173 anode demonstrates a top-rank current density of 2.45 A cm^−2^ at 1.3 V and an extremely low polarization resistance of 0.05 Ω cm^2^ at 650 °C. This multi-scale, multi-faceted research approach not only discovered a high-performance anode but also proved the robust framework for the machine learning-assisted design of anodes for P-SOECs.

**Supplementary Information:**

The online version contains supplementary material available at 10.1007/s40820-025-01764-7.

## Highlights

- Machine learning technique was employed to develop anode for proton-conducting solid oxide electrolysis cells (P-SOEC).
- The screened high-performance La_0.9_Ba_0.1_Co_0.7_Ni_0.3_O_3−*δ*_ (LBCN9173) and La_0.9_Ca_0.1_Co_0.7_Ni_0.3_O_3−*δ*_ (LCCN9173) anodes achieved a synergistic enhancement of water oxidation reaction kinetics and proton-conducting ability.
- P-SOECs with LBCN9173 anode demonstrated a top-rank current density of 2.45 A cm^−2^ and an extremely low polarization resistance of 0.05 Ω cm^2^ at 650 °C.

**Supplementary Information:**

The online version contains supplementary material available at 10.1007/s40820-025-01764-7.

## Graphical abstract

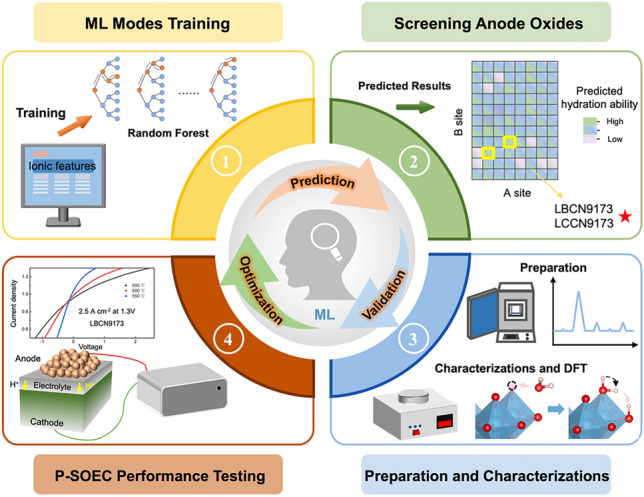

## Introduction

Hydrogen energy has emerged as a promising solution due to its eco-friendliness and high energy density. Current hydrogen energy production technologies operate across various temperature ranges: alkaline water electrolyzers (AWEs) and proton exchange membrane electrolysis cells (PEMECs) at low temperatures (50–90 °C), and oxide-ion conducting and proton-conducting solid oxide electrolysis cells (O-SOECs and P-SOECs) at intermediate-to-high temperatures (400–900 °C) [1]. Among these, AWEs are commercially mature, benefiting from their independence from noble metal catalysts, long-term stability, and low costs. However, they are hindered by high electrode overpotentials, gas crossover, and low current densities (0.2–0.4 A cm^−2^) [2]. PEMWEs, known for their rapid response and high efficiency, are promising for hydrogen generation but remain reliant on noble metal catalysts. In contrast, solid oxide electrolysis cells (SOECs), particularly P-SOECs, are attracting much attention in renewable energy applications for their high electrolysis efficiency (up to 1 A cm^−2^) and cost-effectiveness without the need for noble metal catalysts [2–6]. P-SOECs are especially promising for intermediate temperature (400–700 °C) hydrogen production, offering advantages such as lower activation energy and faster proton migration compared to oxide-ion conducting counterparts. This positions P-SOECs as a superior choice for efficient and sustainable hydrogen energy production.

However, the widespread application of P-SOECs still faces numerous challenges, including electrochemical performance optimization of key materials, interface issues, fabrication of large-scale high-performance cells, etc*.* [2, 6–10]. Developing high-performance anodes for P-SOECs is particularly challenging, which involves the adsorption and dissociation of steam, conduction of oxide ions (O^2−^) and protons, and the transfer of four electrons [2]. Therefore, achieving efficient synergy between the catalytic activity of steam oxidation and ionic/electronic conductivity is the key for designing high-performance anodes. Traditional Co-based perovskite oxides (such as La_1−*x*_Sr_*x*_Co_1−y_Fe_y_O_3−*δ*_ (LSCF) and Ba_1−*x*_Sr_*x*_Co_1−y_Fe_y_O_3−*δ*_ (BSCF)) exhibit good catalytic activity and O^2−^/e^−^ conductivity, called mixed ionic-electronic conductors (MIECs) [11, 12]. However, these conventional materials, such as Ba_0.5_Sr_0.5_Co_0.8_Fe_0.2_O_3−*δ*_, exhibit significantly higher TECs (~ 19–21 × 10^–6^ K^−1^) compared to those (~ 13–16 × 10^–6^ K^−1^) of new Co/Ni-doped La-based perovskites (LaCo_0.2_Cu_0.2_Fe_0.2_Ni_0.2_Cr_0.2_O_3−*δ*_), posing substantial challenges to the stability and performance of P-SOCs [13–20]. In order to make proton conduction and other properties side by side, researchers have adopted various strategies, such as elements dopant, the introduction of proton-conducting phase, and design of oxygen deficiency, to develop oxides with both high ionic/electronic conductivity and catalytic activity, and thus enhancing the electrochemical performances of P-SOECs [2, 11]. For instance, the proton rotation energy of Ba_0.4_K_0.1_Sr_0.5_Co_0.8_Fe_0.2_O_3−*δ*_ was reduced from 0.33 to 0.07 eV by doping BSCF with K [21]. Shao et al. demonstrated that the current density of the P-SOEC increased 3.3 times as the proton-conducting phase Ba_4_Sr_4_(Co_0.8_Fe_0.2_)_4_O_16−*δ*_ introduced to the anode [22]. Ran et al. reported that A-site deficient Ba_0.9_Co_0.4_Fe_0.4_Zr_0.1_Y_0.1_O_3−*δ*_ facilitates the formation of oxygen vacancies, promoting proton conduction [23]. Although some advances have been made in the development of high-efficiency anodes, it is still far from meeting the requirements of commercial P-SOECs. Therefore, further acceleration of research and development is highly needed to rapidly achieve the synergistic enhancement of catalytic activity and ionic conduction.

With the rapid development of artificial intelligence and the continuous enrichment of material databases, machine learning (ML) has shown significant advantages in materials screening. It can efficiently process massive amounts of data, identify complex structure-performance relationships, and accurately predict the properties of unknown materials. Through continuous learning and optimization, ML models constantly improve prediction accuracy, greatly reducing experimental costs and time, accelerating material development cycles, and achieving special designs. It can simultaneously consider multiple parameters, explore new combinations in a vast material space, and promote interdisciplinary knowledge integration. Several research works have demonstrated the effectiveness of ML in material development [24–26]. For example, Ni et al. used the ML model to predict the area-specific resistance of 6871 different perovskite oxides. Among those, Sr_0.9_Cs_0.1_Co_0.9_Nb_0.1_O_3_ oxide was identified and demonstrated exceptional electrochemical activity and low area-specific resistance (ASR) of 0.01 Ω cm^2^ at 700 °C [25]. Liu et al. applied high-throughput calculations and data-driven decomposition analysis to predict the thermodynamic stability of 4,455 distinct perovskite oxides. The selected oxide, PrBaCo_1.9_Hf_0.1_O_5+δ_ shows excellent catalytic activity and stability [27]. Meanwhile, they also reported that BaSn_*x*_Ce_0.8−*x*_Yb_0.2_O_3−*δ*_ electrolyte exhibited superior proton conduction ability compared to the widely used BaZr_0.1_Ce_0.7_Y_0.1_Yb_0.1_O_3−*δ*_ (BZCYYb1711), which is screened by employing high-throughput calculations to predict the vacancy formation energy and hydration reaction energy of 932 electrolytes for P-SOECs [24]. Ye’s group also constructed accurate and interpretable ML models with various algorithms to efficiently screen the anode of solid oxide cells, achieving the current density of 0.88 A cm^−2^ at 600 °C with La_0.7_Ca_0.3_Co_0.8_Ni_0.2_O_3−*δ*_ oxide [28, 29].

In the present study, La_0.9_Ba_0.1_Co_0.7_Ni_0.3_O_3−*δ*_ (LBCN9173) and La_0.9_Ca_0.1_Co_0.7_Ni_0.3_O_3−δ_ (LCCN9173) were tailored using ML model by predicting the hydration proton concentrations (HPCs) of 3200 distinct perovskite oxides. Through experimental methods, such as thermogravimetric analysis (TG), X-ray photoelectron spectroscopy (XPS), and Fourier transform infrared spectroscopy (FT-IR), combined with density functional theory (DFT) calculations on hydration reaction enthalpy, proton migration energy barrier, and catalytic activity, we comprehensively characterized and analyzed the proton uptake and conduction abilities and catalytic activity of two oxides, further validating the reliability of the constructed ML model. Furthermore, we prepared P-SOECs with LBCN9173 and LCCN9173 anodes and conducted their electrochemical performances. The results showed that P-SOECs with LBCN9173 exhibit excellent water electrolysis performances (current density of 2.45 A cm^−2^ at 1.3 V, polarization resistance of 0.05 Ω cm^2^ at 650 °C). Besides, the water oxidation reaction mechanism was elucidated by performing distribution of relaxation times (DRT) and DFT characterizations. Overall, this study has achieved three significant outcomes. First, it reports a novel high-performance anode material that demonstrates excellent electrochemical performance in P-SOECs. Second, it validates the high accuracy and reliability of RF model through comprehensive experimental and DFT calculating verification, establishing a robust approach for materials prediction. Third, it enriches the materials database for P-SOECs through systematic characterization and performance evaluation, providing valuable data resources for accelerating the development of next-generation energy storage and conversion devices.

## Experimental Section

### Materials Synthesis

La_0.9_Ba_0.1_Co_0.7_Ni_0.3_O_3_ (LBCN9173), La_0.9_Ca_0.1_Co_0.7_Ni_0.3_O_3_ (LCCN9173), La_0.9_Sr_0.1_Co_0.7_Ni_0.3_O_3_ (LSCN9173), and LaCo_0.7_Ni_0.3_O_3_ (LCN73) oxides were synthesized following a sol–gel route method reported elsewhere [29]. First, the La(NO_3_)_2_·6H_2_O (Aladdin, 99.99%), Ba(NO_3_)_2_ (Aladdin, 99.99%), Co(NO_3_)_2_·6H_2_O (Aladdin, 99.99%), Ni(NO_3_)_2_·6H_2_O (Aladdin, 98%), Sr(NO_3_)_2_ (Aladdin, 99.97%), and CaCO_3_ (Aladdin, 99.99%) were completely dissolved into secondary water, and the citric acid (Aladdin, 99.5%) was added to the obtained solution at 60 °C. Next, the citrate solution was heated with vigorous stirring at a temperature below 85 °C until the gel formed. Instantly, the gel was heated up to 500 °C for 1 h to remove the polymeric chelate. Finally, LBCN9173, LCCN9173, LSCN9173, and LCN73 oxides were obtained by annealing the precursor in a tube furnace at 900 °C for 8 h in the air.

### Preparation of Cells

#### Single Cell

P-SOEC was fabricated using a solid-state reactive sintering method. Stoichiometric amounts of BaCO_3_ (Aladdin, 99.8%), ZrO_2_ (Aladdin, 99.99%), CeO_2_ (Aladdin, 99.9%), Y_2_O_3_ (Aladdin, 99.999%), and Yb_2_O_3_ (Aladdin, 99.9%) were mixed by ball milling at 350 r min^−1^ with ethyl alcohol as media for 5 h, followed by drying and sintering at 1100 °C in air for 10 h. The cathode was composed of BaZr_0.4_Ce_0.4_Y_0.1_Yb_0.1_O_3−δ_ (BZCYYb4411), NiO (Aladdin, 99%), and starch (Aladdin, 99%) at the weight ratio of 4:6:1. The cathode was pressed into a pellet as a supporter. The electrolyte slurry was then prepared by dispersing in a dispersant (20 wt% polyethyleneimine (MW 28000) dissolved in α-terpineol) and a binder (5 wt% surfactant dissolved in α-terpineol). This slurry was spin-coated onto both surfaces of the cathode pellet to form the electrolyte layers. Subsequently, the cathode and the electrolyte were co-sintered at 1400 °C for 8 h in the air. One side of the disk was polished and painted Ag for the current collection, the other side was screen-printed LBCN9173 or LCCN9173 anode.

#### Symmetrical Cell

BZCYYb4411 powders with 1 wt.% NiO (Aladdin, 99%) as a sintering aid were dry pressed into pellets using a uniaxial press of 20 MPa for 1 min and then calcined at 1550 °C for 8 h. The anode slurry was prepared by ball milling oxides with α-terpineol in a weight ratio of 1:1.5. The symmetrical cell was fabricated by screen-printed the anodic slurry on both sides of BZCYYb4411 pellet.

### Characterizations

The microstructure of oxides and P-SOECs were measured using a scanning electron microscope (SEM; SU8010, HITACHI) with 3 kV acceleration voltage and field emission transmission electron microscopy (TEM, FEI Talos F200x) with 200 kV acceleration voltage. X-ray powder diffraction (XRD; Rigaku, Ultima IV) was performed by Cu Kα radiation (40 kV, 40 mA) in the 2θ range of 20–80°. The surface element states were identified by X-ray photoelectron spectroscopy (XPS, Thermo Scientific ESCALAB 250XI) under dry condition, which used Al Kα (hv = 1486.6 eV) as the source of X-ray, and the beam spot was 650 µm. The voltage and current are 14.8 kV and 1.6 A, respectively. Peak of C 1*s* (284.8 eV) was used for the peak correction. The oxygen vacancies of oxides were qualitatively identified by electron paramagnetic resonance (EPR, Bruker EPR A300-10/12) at room temperature. Fourier transform infrared spectroscopy (FT-IR) measurements were tested at room temperature and dry air by an equipment (TENSOR II + Hyperion2000). The electrical conductivity of oxide was measured by the DC four-probe method. The thermal expansion coefficient (TEC) were performed using PCY-G-1000 in air with a heating rate of 5 °C min^−1^. Thermogravimetric (TG) analysis of LBCN9173 and LCCN9173 oxides was performed by a STA6000 thermogravimetric analyzer (PerkinElmer) at a rate of 5 °C min^−1^ under a mixing flow of dry and wet synthetic air (*p*_H2O_ = 0.02 atm) at 20 sccm. The wet synthetic air was obtained by passing 20% Vol. O_2_/80% Vol. Ar mixed gas at total flow of 20 sccm through a water bubbler at room temperature.

For P-SOEC tests, wet hydrogen (3%H_2_O/H_2_) and wet synthetic air (20% Vol. O_2_/80% Vol. Ar) at total flow rate of 15 and 20 sccm were flowed to cathode and anode, respectively. Silver glue (DAD-87, Shanghai, China) was selected as the sealing material to prevent the gas crossover. The P-SOEC single cell is a circular disk with a diameter of approximately 13 mm, and the effective area of the anode is about 0.12 cm^2^. The electrochemical impedance spectra (EIS) of the P-SOECs were obtained with a CORRTEST frequency response analyzer implemented with a CORRTEST CS2350M potentiostat using the frequency ranging from 10^5^ to 0.1 Hz with an AC amplitude of 30 mV under open circuit voltage (OCV) condition. Moreover, the current–voltage (*I–V*) characteristics were recorded on the same apparatus. Symmetric cell was measured at wet synthetic air with the flow rate of 20 sccm. The DRT converted from impedance spectra was developed by MATLAB.

### DFT Calculation

We employed the Vienna Ab initio Simulation Package (VASP) to perform all DFT calculations with generalized gradient approximation (GGA) using the Perdew–Burke–Ernzerhof (PBE) function. Specifically, projected augmented wave (PAW) potentials were selected to describe the ionic cores. The valence electrons were considered using a plane-wave basis set with a kinetic energy cut-off of 450 eV. Thereafter, geometry optimizations were performed with a force convergence of less than 0.05 eV Å^−1^, where the same convergence was applied to locate the transition states through constrained optimizations (NEB). The oxygen vacancy formation energy (${\Delta}{\text{E}}_{\text{FE}}$), hydration energy, and proton-conducting barrier energy were investigated under spin-polarized calculations on an LBCN9173/LCCN9173 bulk structure containing 120 atoms. The oxygen vacancy was created by deleting a single oxide ion between Co–Co, Co–Ni, and Ni–Ni, written as Co–${\text{V}}_{\text{O}}^{\cdot\cdot}$–Co, Co–${\text{V}}_{\text{O}}^{\cdot\cdot}$–Ni, and Ni–${\text{V}}_{\text{O}}^{\cdot\cdot}$–Ni. Moreover, oxygen evolution reaction (OER) catalytic activity was calculated on the (001) surface of LBCN9173 and LCCN9173 oxides.

${\Delta}{\text{E}}_{\text{FE}}$ of LBCN9173 and LCCN9173 oxides are calculated according to Eq. 1:

1 $${\Delta}{\text{E}}_{\text{FE}}= {\text{E}}_{\text{dehyd}} \, + \frac{1}{{2}}{\text{E}}_{{\text{O}}_{2}} - {{\text{E}}}_{\text{perfect}}$$

Here, ${\text{E}}_{\text{dehyd}}$ and ${\text{E}}_{\text{perfect}}$ are the total energy of defective bulk with ${\text{V}}_{\text{O}}^{\cdot\cdot}$ and perfect bulk, respectively. ${\text{E}}_{{\text{O}}_{2}}$ represents the energy of oxygen molecular in vacuum.

### Machine-Learning Model

Random forest algorithm was employed to predict the HPCs using a comprehensive dataset of 795 perovskite oxides with 66 features, and the data is collected from public references. The model was trained using the "caret" package (short for Classification and Regression Training) in the R platform. Then a tenfold cross-validation resampling procedure was used to improve the model training with the hyperparameter settings of min.node.size = 3, splitrule = beta, and mtry = 24. The model's predictive capability was evaluated through multiple metrics including R^2^, RMSE, and MAE. After assessing the predictive performance including accuracy and interpretability, the RF model was used to predict the unknown HPCs of 3200 oxides.

## Results and Discussion

### Screen the Target Anode Oxides

**Fig. 1:**
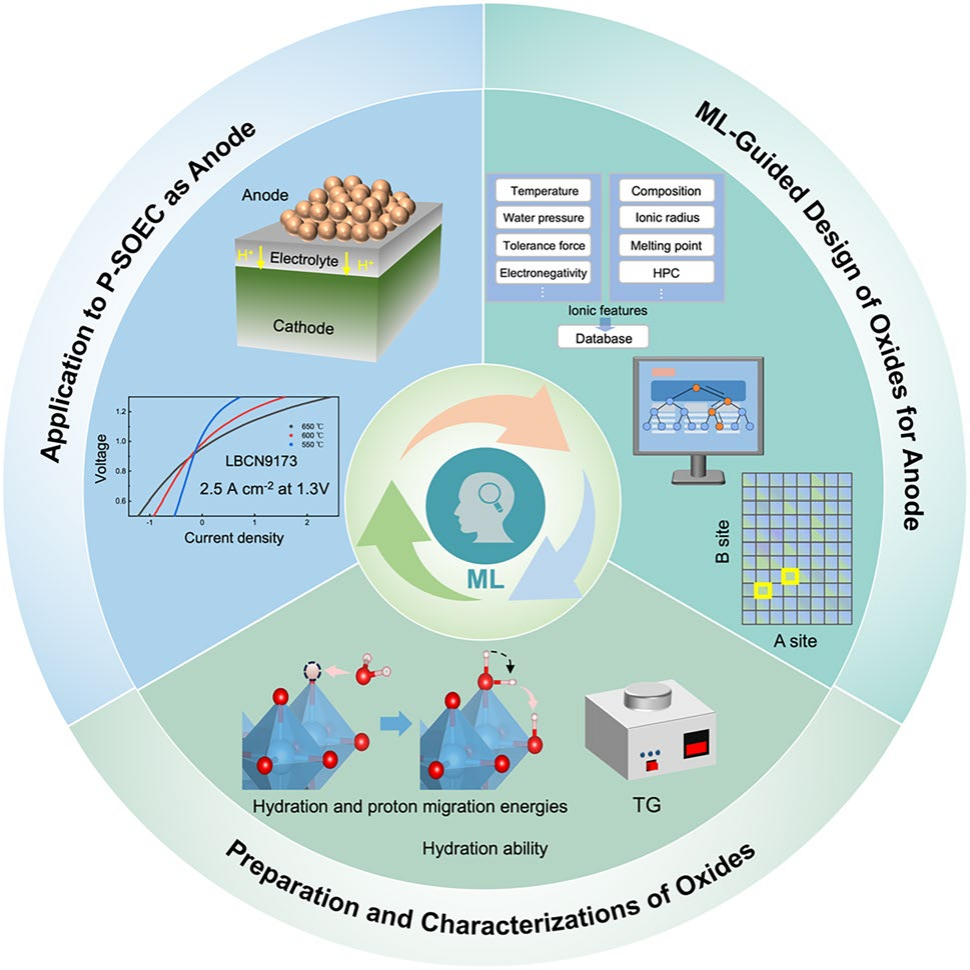
Comprehensive looped workflow: from ML-guided oxide design to experimental validation and performance evaluation in P-SOECs

Figure 1 presents a comprehensive looped workflow for screening and researching high-efficiency anode oxides. This workflow comprises three main parts: ML-guided oxide design, experimental validation, and performance evaluation in P-SOECs, forming an iterative feedback loop. Specifically, ML model is well trained to predict hydrated proton concentration (HPCs) using the collected dataset, which can be used to represent proton conduction ability partly [28–30]. Promising oxide compositions are identified and selected for synthesis and characterization based on the predictions. The experimental results provide crucial feedback, guiding the refinement of the ML models and expanding the dataset. This cycle continuously improves the predictive power of the models while efficiently directing experimental efforts toward the most promising anode oxides for P-SOECs.

**Fig. 2:**
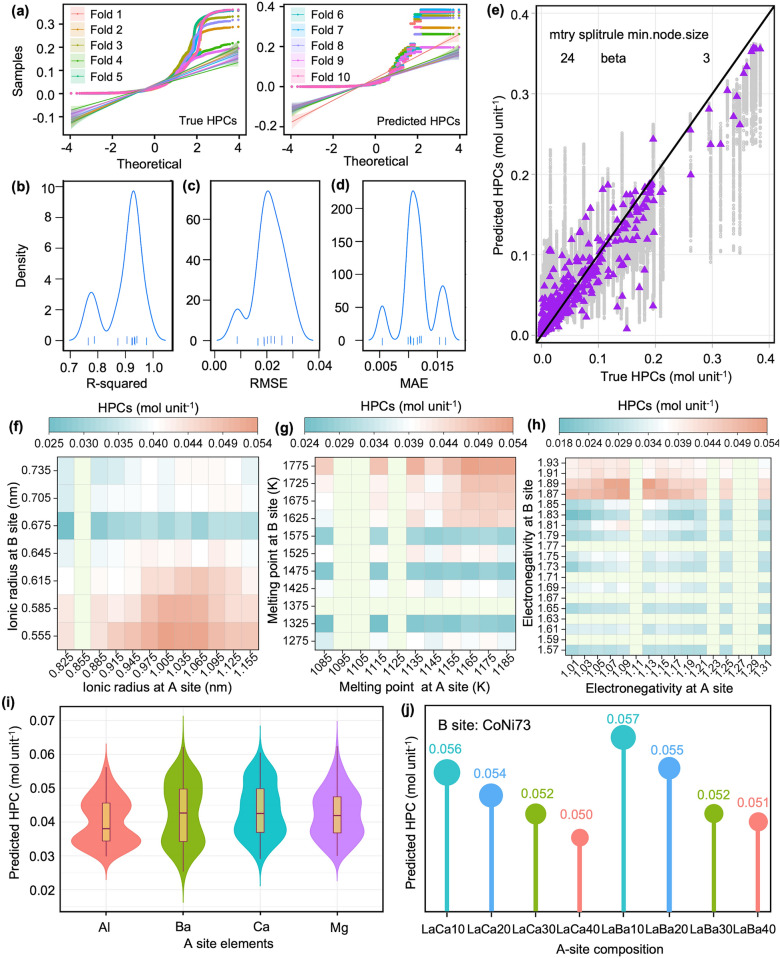
Performance evaluation of ML model and predicting results. **a** Distribution of true and predicted HPCs across tenfold cross-validation. Distribution of **b** R-squared, **c** Root Mean Square Error (RMSE) and **d** Mean Absolute Error (MAE). **e** Scatter plot of true vs. predicted HPCs during the ML training process. **f–h** Correlations between ionic radius, electronegativity, and melting point of A and B site elements with predicted HPCs. **i** Violin plots with overlaid box plots for predicted HPCs of Al-, Ba-, Ca-, or Mg-containing oxides at A site. **j** Predicted HPCs of (La_1−*x*_Ba_*x*_)(Co_0.7_Ni_0.3_)O_3_ and (La_1−*x*_Ca_*x*_)(Co_0.7_Ni_0.3_)O_3_ oxides (*x* = 0.1, 0.2, 0.3, 0.4)

The ML model was trained using a dataset comprising the target HPC and 35 descriptors, including temperature, ionic radius, electronegativity, melting point, etc. We employed the random forest (RF) algorithm due to its advantages in handling nonlinear relationships, feature importance ranking, and robustness against overfitting. A tenfold cross-validation approach was implemented during the training process. The distribution of true and predicted HPCs illustrated in Fig. 2a indicates a wide range of values in both the predicted and training datasets, demonstrating the model's capability to handle diverse HPCs. The model demonstrates excellent performance, characterized by low error rates and high predictive accuracy. The distributions of performance metrics with 95% confidence interval (CI) on the basis of the best tuned hyperparameters (mtry = 24, splitrule = beta, and min.node.size = 3) are presented in Fig. 2b–d, including R-squared (0.8967, 95% CI [0.8962, 0.8972]), root mean square Error (RMSE, 0.021 mol unit⁻^1^, 95% CI [0.0205, 0.0214] mol unit⁻^1^), and mean absolute error (MAE, 0.0115 mol unit⁻^1^, 95% CI [0.011, 0.0119] mol unit⁻^1^). The scatter plot in Fig. 2e visualizes the model's predictive accuracy using these optimized hyperparameters. The model reveals that the ionic radius, melting point, and electronegativity have critical effects on HPCs, as illustrated in Fig. 2f–h. For example, elements with larger ionic radius occupying the A site, combined with cations of smaller ionic radius at the B site, tend to promote hydration reactions in the perovskite oxides. Because the larger A-site elements can provide more space for proton rotation, thereby the enhancing proton mobility and conduction ability. The smaller the ionic radius of the B-site cation, the shorter the bond length between it and the lattice oxygen. This results in a stronger electrostatic attraction between the two ions. This stronger bonding makes it easier for the lattice oxygen to accept protons [11]. Overall, the high-accuracy and explainable RF model is well-constructed and used for the further prediction.

The predicted oxides are divided into four categories based on whether the perovskite structure oxides (ABO_3_) contain Al, Ba, Ca, or Mg at the A-site. Figure 2i shows the violin plots with overlaid box plots for HPCs of these oxides. Each dopant is represented by a differently colored violin shape, providing a visual representation of the data distribution. Both Ba-(0.042 mol unit^−1^) and Ca-(0.042 mol unit^−1^) containing oxides show higher median predicted HPCs compared to Al-(0.038 mol unit^−1^) and Mg-(0.041 mol unit^−1^) containing oxides, and their distributions extend to higher values, indicating the potential for high HPCs. The box plots within these violins also suggest a wider interquartile range for Ba-containing oxides compared to Ca-containing oxides, further emphasizing the high-HPCs possibility of Ba-containing oxides. The results indicate that Ba and Ca at the A-site are conducive to proton incorporation, making them ideal proton conduction. Based on our previous report, oxides with Co_0.7_Ni_0.3_ at B-site show promising proton conductivity and water oxidation catalytic ability [29]. Accordingly, (La,Ba)(Co_0.7_Ni_0.3_)O_3_ and (La,Ca)(Co_0.7_Ni_0.3_)O_3_ oxides were selected to be the target oxides. Further, HPCs of (La_1−*x*_Ba_*x*_)(Co_0.7_Ni_0.3_)O_3_ and (La_1−*x*_Ca_*x*_)(Co_0.7_Ni_0.3_)O_3_ oxides (*x* = 0.1, 0.2, 0.3, 0.4) are predicted and shown in Fig. 2j. The results manifest that (La_0.9_Ba_0.1_)(Co_0.7_Ni_0.3_)O_3_ (LBCN9173) and (La_0.9_Ca_0.1_)(Co_0.7_Ni_0.3_)O_3_ (LCCN9173) oxides show the highest HPCs at each oxide family. Finally, LBCN9173 and LCCN9173 oxides are selected for further preparation, characterizations and application.

### Phase Structure and Microstructure of the Screened Oxides

**Fig. 3:**
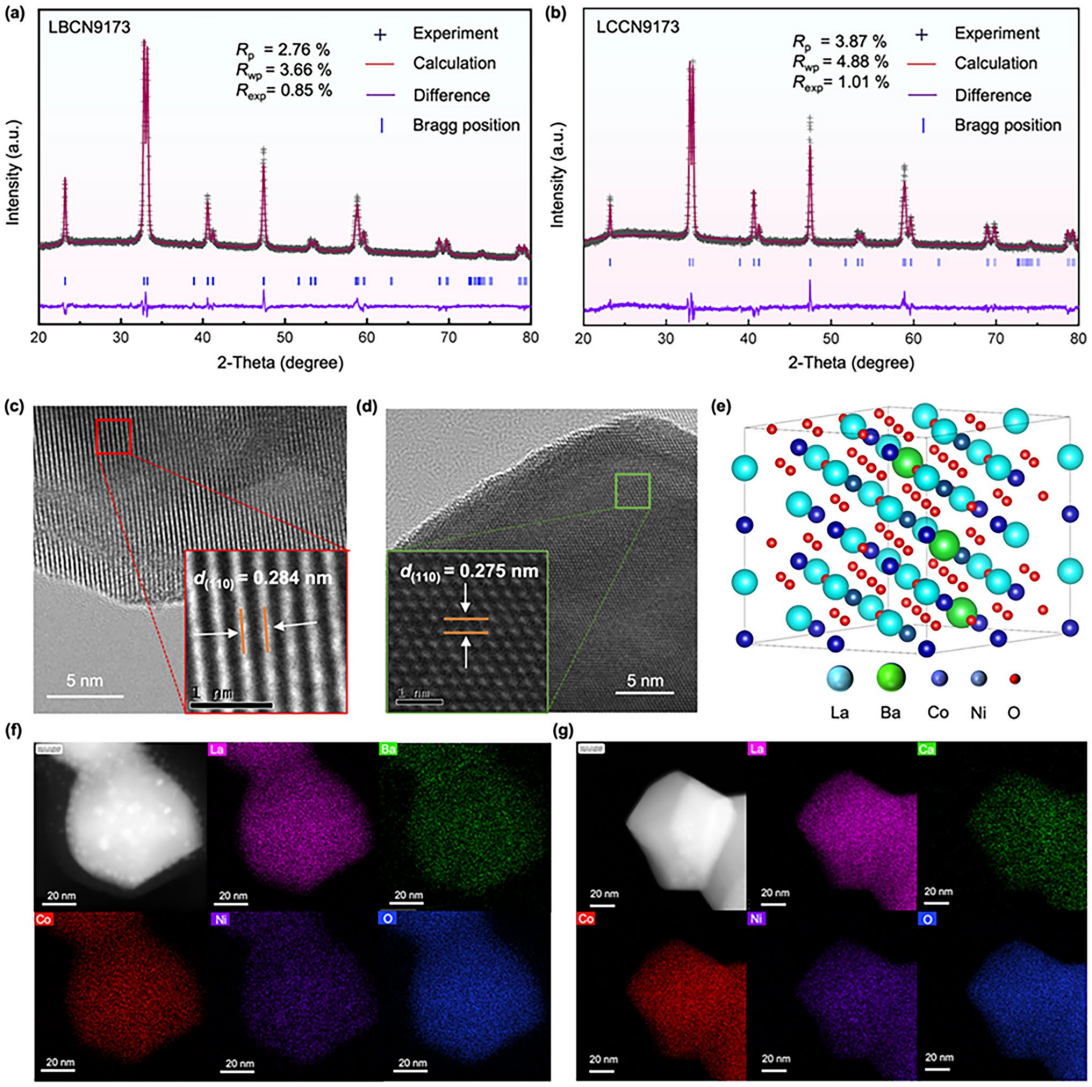
Phase structure and microstructure of LBCN9173 and LCCN9173 oxides. **a, b** XRD refinement results, **c, d** HR-TEM images, and **f, g** STEM-EDX mapping images for LBCN9173 and LCCN9173 oxides. **e** Phase structure of LBCN9173 oxide

X-ray diffraction (XRD) patterns (Figs. 3a, b and S1a) suggest that LBCN9173 and LCCN9173 oxides display a pure perovskite structure without observable secondary phase, corresponds well with the PDF card of LaCoO_3−*δ*_ (${\text{R}}\stackrel{\text{-}}{3}{\text{c}}$, PDF # 48-0123). The (110) peak at 32°–34° of LBCN9173 oxide (Fig. S1b) shifts to lower angle compared to that of LCCN9173 oxide, indicating an expansion of LBCN9173 oxide lattice. The XRD Rietveld refinements were conducted with rhombohedral (${\text{R}}\stackrel{\text{-}}{3}{\text{c}}$) LaCoO_3−*δ*_-derived phase structure, shown in Figs. 3e and S2. The results are reliable with small *R*_p_ (2.76% and 3.87%),* R*_wp_ (3.66% and 4.88%) and *R*_exp_ (0.85% and 1.01%) for LBCN9173 and LCCN9173 oxides. The calculated lattice parameters of LBCN9173 (5.4547 Å) oxide is larger than that of LCCN9173 (5.4476 Å) (Table S1) oxide. High resolution transmission electron microscope (HR-TEM) images also suggest the expanded lattice of LBCN9173 oxide with the (110) interplanar spacing of 0.284 nm, higher than that of LCCN9173 oxide (0.275 nm) (Fig. 3c, d). These phenomena are reasonable due to the larger ionic radius of Ba^2^⁺ (0.135 nm) than Ca^2^⁺ (0.100 nm). Figure 3f, g shows the elemental distribution of LBCN9173 and LCCN9173 oxides, indicating the homogeneous distribution of elements La, Ba/Ca, Co, Ni, and O without any element segregation, and it also suggests that Ba and Ca have been successfully incorporated into the LBCN9173 and LCCN9173 oxides lattice, respectively.

### Proton Uptake and Conduction Ability of the Screened Oxides

**Fig. 4:**
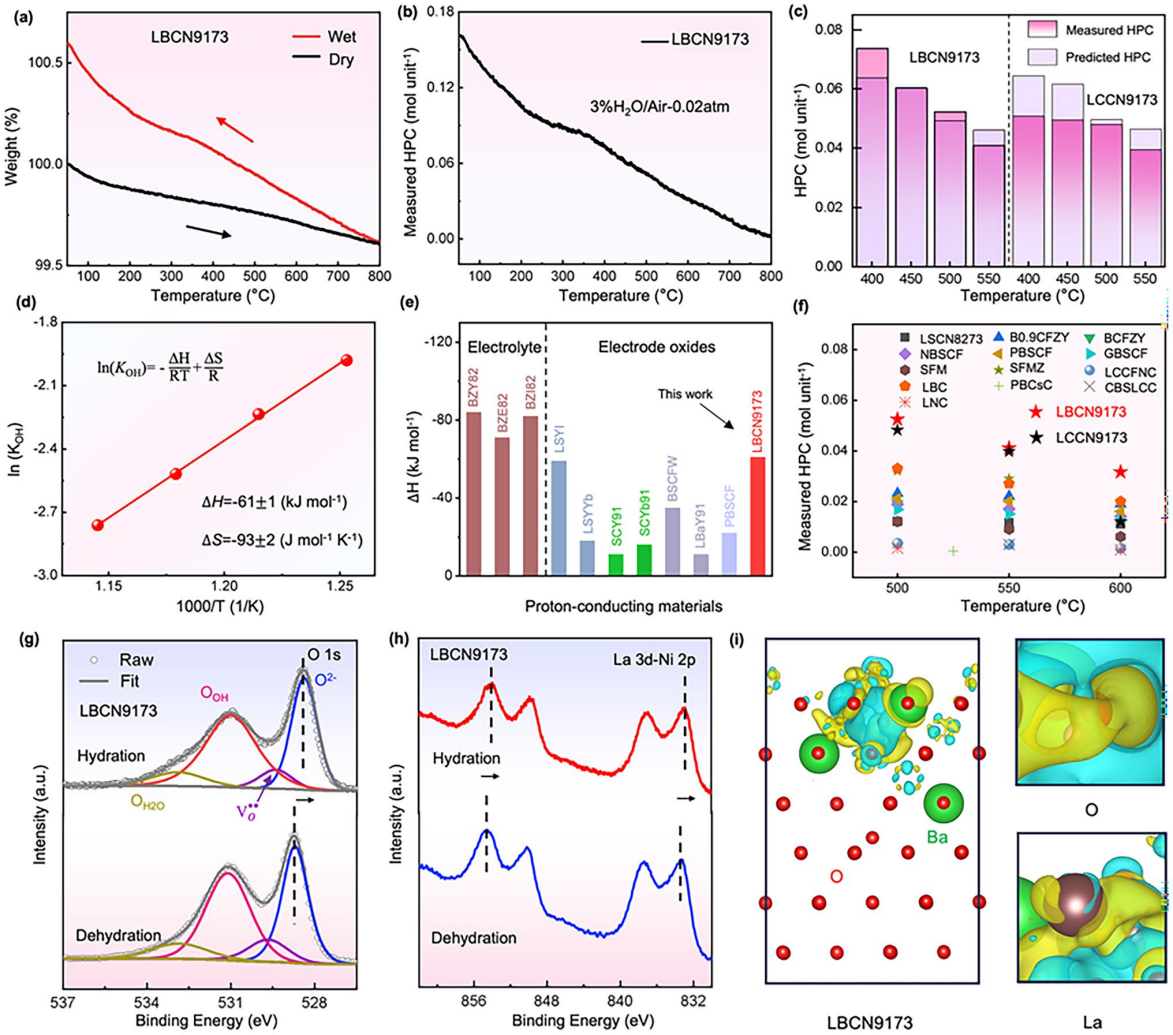
**a** TG curves of LBCN9173 oxide under dry and wet air (*p*_H2O_ = 0.02 atm) at 50–800 °C. **b** Measured HPCs of LBCN9173 oxide. **c** Comparison of measured and predicted HPCs at 400, 450, 500, and 550 °C for LBCN9173 and LCCN9173 oxides. **d** Arrhenius plot showing the equilibrium constant of hydration reaction as a function of temperature for LBCN9173 oxide. **e** Δ*H* of LBCN9173 oxide and reported oxides. **f** Summary of HPCs for LBCN9173, LCCN9173, and reported oxides. XPS spectra of **g** O 1*s* and **h** La 3*d*-Ni 2*p* for hydrated and dehydrated LBCN9173 samples. **i** The differential charge density of hydrated LBCN9173 oxide with two protons. Abbreviations: BZY82 = BaZr_0.8_Y_0.2_O_3−δ_ [31], BZI82 = BaZr_0.8_In_0.2_O_3−δ,_ BZE82 = BaZr_0.8_Er_0.2_O_3−δ_, LSYI = La_0.9_Sr_0.1_Y_0.8_In_0.2_O_3−δ_, SCY91 = SrCe_0.9_Y_0.1_O_3−δ_, SCYb91 = SrCe_0.9_Yb_0.1_O_3−δ_, LSYYb = La_0.9_Sr_0.1_Y_0.8_Yb_0.2_O_3−δ_ [32], BSCFW = Ba_0.5_Sr_0.5_(Co_0.7_Fe_0.3_)_0.6875_W_0.3125_O_3−δ_ [33], LBYb91 = La_0.9_Ba_0.1_YbO_3−δ_ [34], PBSCF = PrBa_0.5_Sr_0.5_Co_1.5_Fe_0.5_O_5+δ_ [35], SFM = Sr_2_Fe_1.5_Mo_0.5_O_6−δ_, SFMZ = Sr_2_Fe_1.5_Mo_0.4_Zr_0.1_O_6−δ_ [36], GBSCF = GdBa_0.5_Sr_0.5_Co_1.5_Fe_0.5_O_5+δ_, NBSCF = NdBa_0.5_Sr_0.5_Co_1.5_Fe_0.5_O_5+δ_ [37], BCFZY = BaCo_0.4_Fe_0.4_Zr_0.1_Y_0.1_O_3−δ_ [38], B0.9CFZY = Ba_0.9_Co_0.4_Fe_0.4_Zr_0.1_Y_0.1_O_3−δ_ [23], LSCN8273 = La_0.8_Sr_0.2_Co_0.7_Ni_0.3_O_3−δ_ [39], LBC = La_0.9_Ba_0.1_CoO_3−δ_ [40], LCCFNC = La(Co_0.2_Cu_0.2_Fe_0.2_Ni_0.2_Cr_0.2_)O_3−δ_ [14], CBSLCC = Ce_0.2_Ba_0.2_Sr_0.2_La_0.2_Ca_0.2_CoO_3−δ_ [41], PBCsC = PrBa_0.875_Cs_0.125_Co_2_O_5+δ_ [42], LNC = La_2_Ni_0.9_Co_0.1_O_4+δ_ [43]

Figures 4a and S3 show the TG curves of LBCN9173 and LCCN9173 oxides under dry and wet (*p*_H2O_ = 0.02 atm) air at 50–800 °C. A distinct weight gap (defined as ΔW) appears between the two curves due to the hydration reaction as described in Kr$\ddot{\text{o}}$ger–Vink notation (Eq. 2) [44]. For example, the ΔW for LBCN9173 and LCCN9173 oxides is 0.270% and 0.197% at 500 °C, respectively. The measured HPCs of LBCN9173 and LCCN9173 oxides can be quantified by using the ΔW, which are the twice as the absorbed water molar mass (HPC = 2 × Δ*n* (H_2_O)/Δ*n* (oxide)), shown in Figs. 4b and S4 [12]. The measured HPCs of LBCN9173 oxide are 0.060, 0.052, and 0.041 mol unit^−1^ at 450, 500, and 550 °C, respectively, higher than those of LCCN9173 oxide (0.050, 0.048, and 0.039 mol unit^−1^). Moreover, the experimental results match well with the predicted HPCs (Fig. 4c). For instance, the measured *vs.* predicted HPCs of LBCN9173 oxide are 0.052 *vs.* 0.049 mol unit^−1^ and 0.041 *vs.* 0.046 mol unit^−1^ at 500 and 550 °C, respectively. The results suggest that the used ML model is highly accurate and reliable. Furthermore, HPCs of LBCN9173 and LCCN9173 oxides as function of *p*_H2O_ (0–0.05 atm) and temperatures (50–800 °C) are further predicted, shown in Fig. S5, offering a guideline for the application of these oxides in wide ranges of steam pressure and temperature. The HPCs of LBCN9173 oxide are higher than 0.04 mol unit^−1^ above 500 °C, which are superior to many other reported anodes (Fig. 4f), such as BaCo_0.4_Fe_0.4_Zr_0.1_Y_0.1_O_3−δ_ (0.019 mol unit^−1^) [38], PrBa_0.5_Sr_0.5_Co_1.5_Fe_0.5_O_5+δ_ (0.02 mol unit^−1^) [35], enabling the matchable proton conduction in-between the electrolyte and the anode during steam electrolysis [14, 35–43].

The excellent hydration ability of LBCN9173 oxide drives us to understand its thermodynamics. The equilibrium constant of hydration (*K*_OH_), hydration enthalpy (Δ*H*) and hydration entropy (Δ*S*) are calculated. Note that the sum of concentration of ${\text{V}}_{\text{O}}^{\cdot\cdot}$, ${\text{OH}}_{\text{O}}^{\cdot}$ and ${\text{O}}_{\text{O}}^{\times }$ is 3 in ABO_3_ perovskite oxides. Combined with the known parameters, *K*_OH_ expressed as Eq. 3 can be calculated [35]. Shown in Fig. 4d is the Arrhenius plot for *K*_OH_ as function of temperature. Accordingly, Δ*H* and Δ*S* of the oxide are − 61 ± 1 kJ mol^−1^and − 93 ± 2 J mol^−1^ K^−1^ (Fig. 4d) based on the Van't H$\ddot{\text{o}}$ff equation (Eq. 4) [33]. The more negative values of Δ*H* indicate that hydration reaction is more favorable. Δ*H* of LBCN9173 oxide is lower than the well-known anodes, such as Ba_0.5_Sr_0.5_(Co_0.7_Fe_0.3_)_0.6875_W_0.3125_O_3−δ_ (− 35 kJ mol^−1^) [33], PrBa_0.5_Sr_0.5_Co_1.5_Fe_0.5_O_5+δ_ (− 22 kJ mol^−1^ at 400 °C) [35], and even can be comparable to these of the electrolytes (Fig. 4e) [31–35]. Overall, LBCN9173 selected through the ML model exhibits the excellent hydration ability.

2 $${\text{O}}_{\text{O}}^{\times } + {\text{V}}_{\text{O}}^{\cdot\cdot} + {\text{ H}}_{2}\text{O }\to \text{ 2}{\text{OH}}_{\text{O}}^{\cdot}$$

3 $$K_\text{OH}=\frac{{\left[{\text{OH}}_{\text{O}}^{\cdot}\right]}^{2}}{{\text{p}}_{\text{H2O}}\left[{\text{V}}_{\text{O}}^{\cdot\cdot}\right][{\text{O}}_{\text{O}}^{x}]}$$

4 $$\text{ln}\left(K_\text{OH}\right)= -\frac{{\Delta}{\text{H}}}{\text{RT}} + \frac{{\Delta}{\text{S}}}{\text{R}}$$

where ${\text{V}}_{\text{O}}^{\cdot\cdot}$, ${\text{OH}}_{\text{O}}^{\cdot}$ and ${\text{O}}_{\text{O}}^{\times }$ represent oxygen vacancies, proton defect, and lattice oxygen in Eqs. 2 and 3, respectively. *R* and *T* represent the universal gas constant and absolute temperature in Eq. 4.

In order to characterize the proton defects, LBCN9173 oxide was treated at 500 °C under dry and wet air for 3 h, respectively, called dehydrated and hydrated LBCN9173 samples, then immediately measured using the XPS and FT-IR. FT-IR spectra of hydrated LBCN9173 oxide present a prominent hydroxyl (–OH) peak at approximately 3500 cm^−1^ compared to the dehydrated one (Fig. S6) [45]. This further confirms that hydration reaction occurs in LBCN9173 oxide. The high-resolution narrow XPS spectra of O 1*s*, Co 2*p*, La 3*d*-Ni 2*p*, and Ba 3*d* for the hydrated and dehydrated LBCN9173 samples are present in Figs. 4g, h and S7. The O 1*s* XPS spectra are deconvoluted into four peaks at approximately 528.3, 529.3, 530.9, and 532.9 eV, corresponding to lattice oxygen (O^2−^), chemisorbed oxygen at oxygen vacancy (${\text{V}}_{\text{O}}^{\cdot\cdot}$) site, hydroxylated oxygen species (O_OH_), and absorbed surface water (O_H2O_), respectively [46]. The fitting results are shown in Table S2. The ratio of ${\text{V}}_{\text{O}}^{\cdot\cdot}$/O^2−^ decreases from 27 to 21%, and the O_OH_ increases from 44 to 48% after hydration, indicating the occurrence of proton defects in LBCN9173 oxide (Fig. S8) [44]. Moreover, the O 1*s* XPS fitting results revealed that LBCN9173 exhibits a higher ${\text{V}}_{\text{O}}^{\cdot\cdot}$ concentration than that of LCCN9173 (Fig. S8 and Table S2), which is also evidenced by the stronger EPR signal intensity of LBCN9173 compared to that of LCCN9173 (Fig. S9), indicating a higher ${\text{V}}_{\text{O}}^{\cdot\cdot}$ concentration [47]. As compared with LaCo_0.7_Ni_0.3_O_3_ (LCN73) with rhombohedral phase structure (Fig. S10), LBCN9173 and LCCN9173 possess more oxygen vacancy due to the acceptor doping at A-site [21, 48]. When acceptor doping is introduced at the A-site, it fundamentally leads to either an increase in hole conductivity or a rise in ${\text{V}}_{\text{O}}^{\cdot\cdot}$ concentration. The electrical conductivity of LBCN9173 ranges from 120 to 304 S cm^−1^ at 350–750 °C, lowering than those of LaCoO_3_ (430–675 S cm^−1^) and La_0.95_Ba_0.05_CoO_3_ (300–360 S cm^−1^), shown in Fig. S11. The above results indicate that LBCN9173 tends to increase the ${\text{V}}_{\text{O}}^{\cdot\cdot}$ concentration rather than the hole conductivity to maintain charge neutrality as Ba doped at A-site.

In addition, it is evident that the charge accumulation around lattice oxygen attributes to the excellent ability of LBCN9173 oxide (Fig. 4i), making the proton a more favorable attachment. In Fig. 4i, the yellow region represents charge accumulation, whereas the blue region represents charge reduction. There is apparent charge accumulation around lattice oxygen and La, which corresponds well with the negative shift of O 1*s* and La 3*d* spectra (Fig. 4g, h). As for LCCN9173 oxide, peak shift of all elements and charge accumulation cannot be observed during the hydration reaction (Figs. S12 and S13), ascribing the smaller ionic radius of Ca^2+^ (0.100 nm) than that of Ba^2+^ (0.135 nm) [47, 49, 50].

### DFT Calculations on Proton-Conducting Ability and Catalytic Activity

**Fig. 5:**
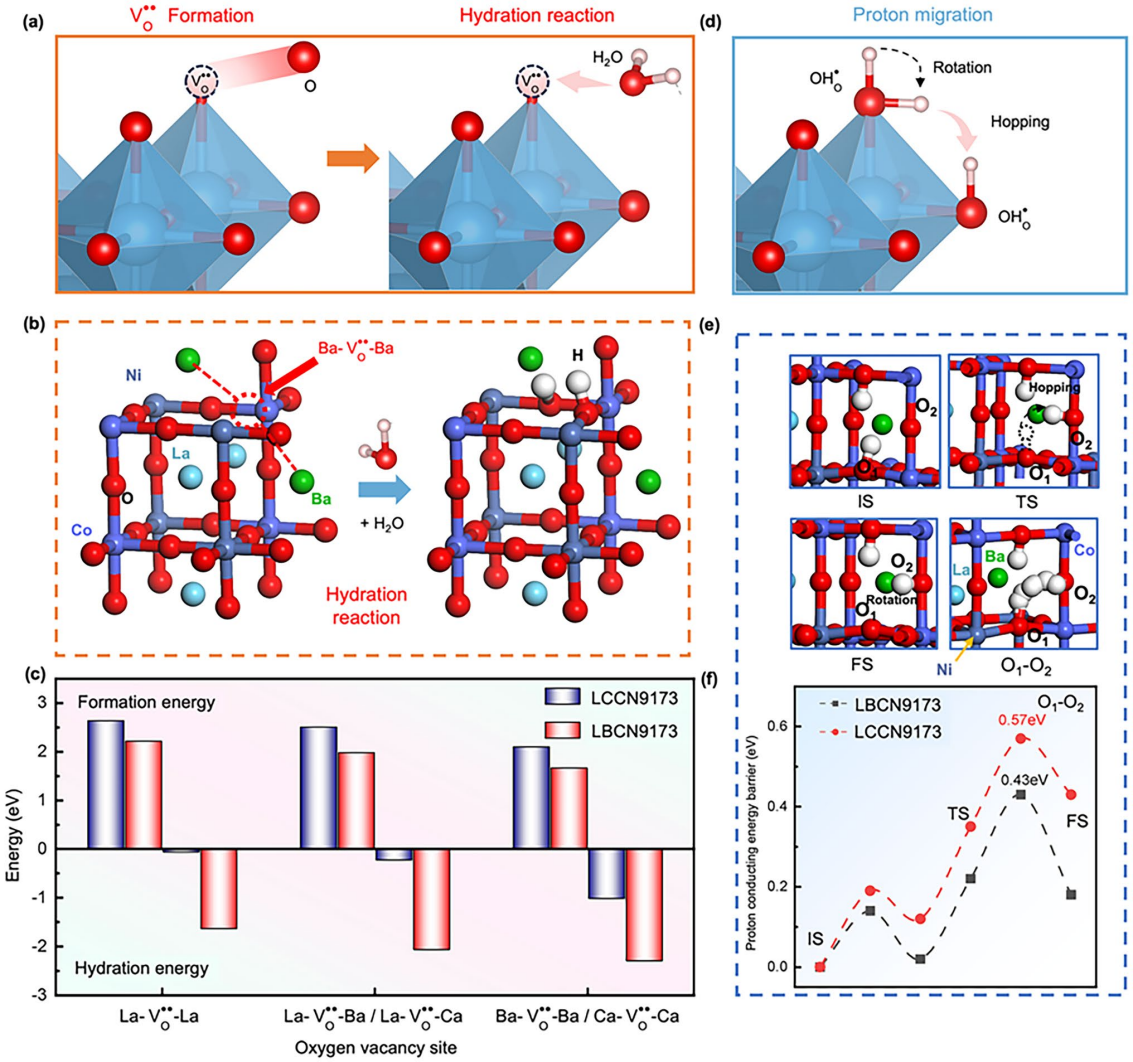
Scheme of **a** ${\text{V}}_{\text{O}}^{\cdot\cdot}$ formation, hydration reaction and **d** proton rotation / hopping in perovskite oxides. **b** Models for hydration reaction process of LBCN9173 oxide. **c** Calculated ${\text{V}}_{\text{O}}^{\cdot\cdot}$ formation energy and hydration energy of LBCN9173 and LCCN9173 oxides with ${\text{V}}_{\text{O}}^{\cdot\cdot}$ at La-La site, Ba/Ca-La site, and Ba/Ca-Ba/Ca site. **e** Models (IS, TS, and FS) for proton transferring from O_1_ to O_2_ site. **f** Proton-conducting energy barriers from O_1_ to O_2_ site in LBCN9173 and LCCN9173 oxides

To further investigate the proton conduction ability of LBCN9173 and LCCN9173 oxides, we performed DFT calculations on their ${\text{V}}_{\text{O}}^{\cdot\cdot}$ formation energies (${\Delta}{\text{E}}_{\text{FE}}$), hydration energy (${\Delta}{\text{E}}_{\text{hydration}}$) and proton-conducting energy barriers. Structure models containing 120 atoms were constructed, as illustrated in Figs. 3e and S2. The ${\text{V}}_{\text{O}}^{\cdot\cdot}$ was created by extracting lattice oxygen neighboring the La–La, Ba/Ca–La, and Ba/Ca–Ba/Ca sites from the perfect lattice (Figs. 5a, b and S14, S15). The calculated ${\Delta}{\text{E}}_{\text{FE}}$ for LBCN9173 oxide are 2.23 eV at the La–La site, 1.99 eV at the Ba–La site, and 1.67 eV at the Ba–Ba site. These values are all lower than their counterparts in LCCN9173 oxide (Fig. 5c and Table S3), indicating that LBCN9173 oxide is more favorable for the formation of ${\text{V}}_{\text{O}}^{\cdot\cdot}$. These values are all lower than those of LCCN9173 oxide (Fig. 5c and Table S3), indicating that LBCN9173 oxide is more favorable for the formation of ${\text{V}}_{\text{O}}^{\cdot\cdot}$. The calculated results are in consistent with the experimental results (Figs. S8 and S9), that is the lower ${\Delta}{\text{E}}_{\text{FE}}$ indicates the higher ${\text{V}}_{\text{O}}^{\cdot\cdot}$ concentration. This enhanced propensity for ${\text{V}}_{\text{O}}^{\cdot\cdot}$ formation in LBCN9173 can be attributed to the larger ionic radius of Ba^2+^ (0.135 nm) compared to Ca^2+^ (0.100 nm) [21]. Because the presence of larger Ba^2+^ ions induces several structural and electronic effects, such as increased steric hindrance, enhanced lattice relaxation and stress release, increased bond length and reduced bond energy, and changes in charge distribution and electronic structure [21, 51, 52]. These factors collectively contribute to the lower ${\text{V}}_{\text{O}}^{\cdot\cdot}$ formation energy, and thus the higher ${\text{V}}_{\text{O}}^{\cdot\cdot}$ concentration in LBCN9173 oxide (Figs. 5c and S8, S9, and Table S3).

To model the hydration process, a proton defect and a proton split from water are incorporated into the formed ${\text{V}}_{\text{O}}^{\cdot\cdot}$, with the latter bonding to a lattice oxygen (Fig. 5a). Accordingly, ${\Delta}{\text{E}}_{\text{hydration}}$ can be calculated based on Eq. 5 [28]. The ${\Delta}{\text{E}}_{\text{hydration}}$ calculating results for LBCN9173 and LCCN9173 oxides with three types ${\text{V}}_{\text{O}}^{\cdot\cdot}$ are shown in Figs. 5b and S14, S15, and Table S3. ${\Delta}{\text{E}}_{\text{hydration}}$ of LBCN9173 oxide is consistently lower than that of LCCN9173 oxide (− 2.30 eV at Ba–Ba site and − 1.02 eV at Ca–Ca site), demonstrating the superior hydration capability of LBCN9173 to LCCN9173 oxides. The results correspond with both experimental and predicted HPCs mentioned above.

5 $${\Delta}{\text{E}}_{\text{hydration}} \, = {\text{E}}_{\text{hyd}} \, - { \, {\text{E}}}_{{\text{de}}{\text{hyd}}} - { \, {\text{E}}}_{{\text{H}}_{2}{\text{O}}}$$

where ${\Delta}{\text{E}}_{\text{hydration}}$ is the hydration energy, ${\text{E}}_{\text{hyd}}$ represent the total energy of the defective bulk with protons after the hydration reaction, ${\text{E}}_{{\text{de}}{\text{hyd}}}$ is the total energy of the defective bulk with ${\text{V}}_{\text{O}}^{\cdot\cdot}$, and ${\text{E}}_{{\text{H}}_{2}{\text{O}}}$ is the energy of a single water molecule in a vacuum.

Given that the Ba–Ba (LBCN9173) and Ca–Ca (LCCN9173) sites exhibit the lowest ${\Delta}{\text{E}}_{\text{hydration}}$, we focused our subsequent analysis on this hydrated state to determine the proton-conducting energy barrier. The proton conduction mechanism involves processes such as proton rotation and hopping (Fig. 5d) [11]. For LBCN9173 and LCCN9173, the proton-conducting energy barriers from O1 to O2 are 0.43 and 0.57 eV, respectively, while the barriers from O2 to O3 are 0.74 and 0.79 eV, respectively (Figs. 5f and S16, and Table S4). These results clearly demonstrate that LBCN9173 exhibits consistently lower proton migration energy barriers along the same migration path compared to LCCN9173, confirming its superior proton conduction ability. Evidently, the lower energy barrier observed for the O_1_ to O_2_ migration path suggests that proton migration is more favorable along this route [29]. The initials states (IS), transition state (TS), final states (FS), and the complete paths are presented in Figs. 5e and S17, S18. Moreover, this migration energy barrier (0.43 eV) is comparably to some previously reported proton-conducting oxide (e.g., BSCF (0.55 eV) [21], La_0.5_Sr_0.5_Fe_0.9_Sb_0.1_O_3−δ_ (0.79 eV) [53], La_0.5_Sr_0.5_MnO_3−δ_ (0.684 eV)) [54], BaCeO_3−δ_ (0.46 eV) [55], BaZr_0.85_Y_0.15_O_3−δ_ (0.43 eV), and BaCe_0.85_Y_0.15_O_3−δ_ (0.59 eV) [31].

**Fig. 6:**
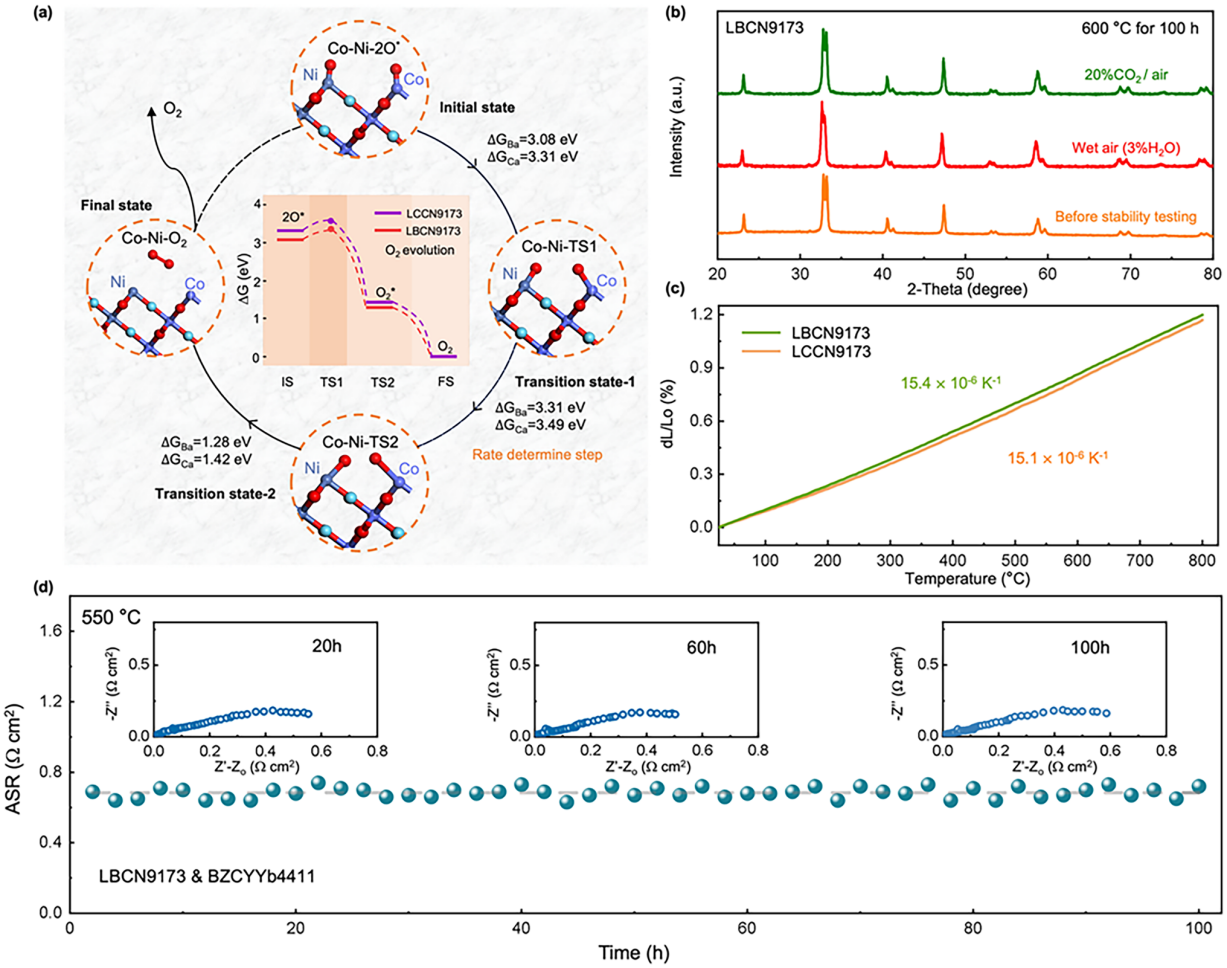
**a** Illustration of OER reaction states (IS, TS1, TS2, FS) and the Gibbs free energy changes profiles of OER at Co–Ni site on (001) surface for LBCN9173 and LCCN9173 oxides. **b** XRD patterns of LBCN9173 before and after exposing to wet air and mixture gas (air with 20% CO_2_), respectively, for 100 h at 600 °C. **c** TEC curves of LBCN9173 and LCCN9173 oxides.** d** ASR stability of the LBCN9173 in wet air at 550 °C

Besides proton conduction ability of LBCN9173 and LCCN9173 oxides, Gibbs free energies for oxygen evolution reaction (OER) processes, which is also a crucial property of anode, were calculated on (001) surfaces of LBCN9173 and LCCN9173 oxides with 120 atoms fixed at the bottom (Figs. 3e and S2). Shown in Figs. 6a and S19, OER processes are divided into several steps, i.e., 1) the initial state (IS) corresponds to the reduction of adsorbed oxygen species (2O^*^), 2) the transition states (TS) involve the adsorption of the oxygen molecule (O_2_^*^), and 3) the final state (FS) represents the formation of molecular oxygen (O_2_) [28]. The Gibbs free energy changes (∆G) of each step is located at the center of Fig. 6a. The OER reaction energy barriers are 3.31 eV and 3.49 eV, respectively, for LBCN9173 and LCCN9173 oxides, indicating the higher OER catalytic activity of LBCN9173 oxide (Fig. 6a). Overall, LBCN9173 oxide shows superior proton-conducting ability and OER catalytic activity to LCCN9173 oxide.

Long-term stability is a critical factor for the anode of P-SOEC. Therefore, the tolerance to steam and CO_2_ for LBCN9173 oxide were tested in wet air (*p*_H2O_ = 0.02 atm) and mixture gas (air with 20% CO_2_), respectively, at 600 °C for 100 h. The XRD patterns of LBCN9173 after these treatments show a pure phase without any change as compared to that of before treatment (Fig. 6b). In addition, the stability of the BZCYYb4411-symmetric cell with LBCN9173 electrode was conducted at 550 °C for 100 h by measuring EIS, shown in Fig. 6d. The ASR is stable at 0.684 Ω cm^2^, indicating the good stability and catalytic activity of LBCN9173 as electrode.

### Application to Anodes of P-SOECs

LBCN9173 and LCCN9173 oxides were employed as anodes of P-SOECs for single-cell testing. XRD patterns (Fig. S20) of the cell components confirm the presence of BZCYYb4411 phase in the electrolyte layer and the presence of NiO-BZCYYb4411 phases in the cathode support, without the impurity phase. The cross-section SEM images of both cells displayed in Figs. S21a and S22a reveal a typical sandwich structure, which is configured with the identical BZCYYb4411 electrolyte (~ 11–13 μm) layer, a porous Ni-BZCYYb4411 cathode support and a porous anode layer (Figs. S21b, c and S22b, c). Figures S21b and S22b provide a magnified view of the interface between the electrolyte and anode, demonstrating the strong bonding between these two layers, which can be attributed to the lower TECs of LBCN9173 (15.4 × 10^–6^ K^−1^) and LCCN9173 (15.1 × 10^–6^ K^−1^) oxides (Fig. 6c). These TEC values are lower than the well-known electrode, such as Ba_0.5_Sr_0.5_Co_0.8_Fe_0.2_O_3−*δ*_ (BSCF, 19.7 × 10^–6^ K^−1^), BaCo_0.4_Fe_0.4_Zr_0.1_Y_0.1_O_3−δ_ (BCFZY, 20.4 × 10^–6^ K^−1^), and PrNi_0.5_Co_0.5_O_3−*δ*_ (PNC, 19.2 × 10^–6^ K^−1^), and even can be comparable with the Co-free oxides, such as La_0.6_Sr_0.4_Fe_0.8_Ni_0.2_O_3−*δ*_ (13.7 × 10^–6^ K^−1^) and Pr_0.5_Sr_0.5_Fe_0.8_Cu_0.2_O_3−*δ*_ (16.4 × 10^–6^ K^−1^), listed in Table S5. Furthermore, when the anodes and BZCYYb4411 composite (with a mass ratio of 1:1) were calcined at 650 °C in air for 50 h, the XRD patterns revealed that the individual phases were maintained without the appearance of additional peaks (Fig. S23). This confirms the excellent stability of the constructed cell and the electrolyte/electrode interfaces, as well as the absence of any undesirable chemical reactions.

**Fig. 7:**
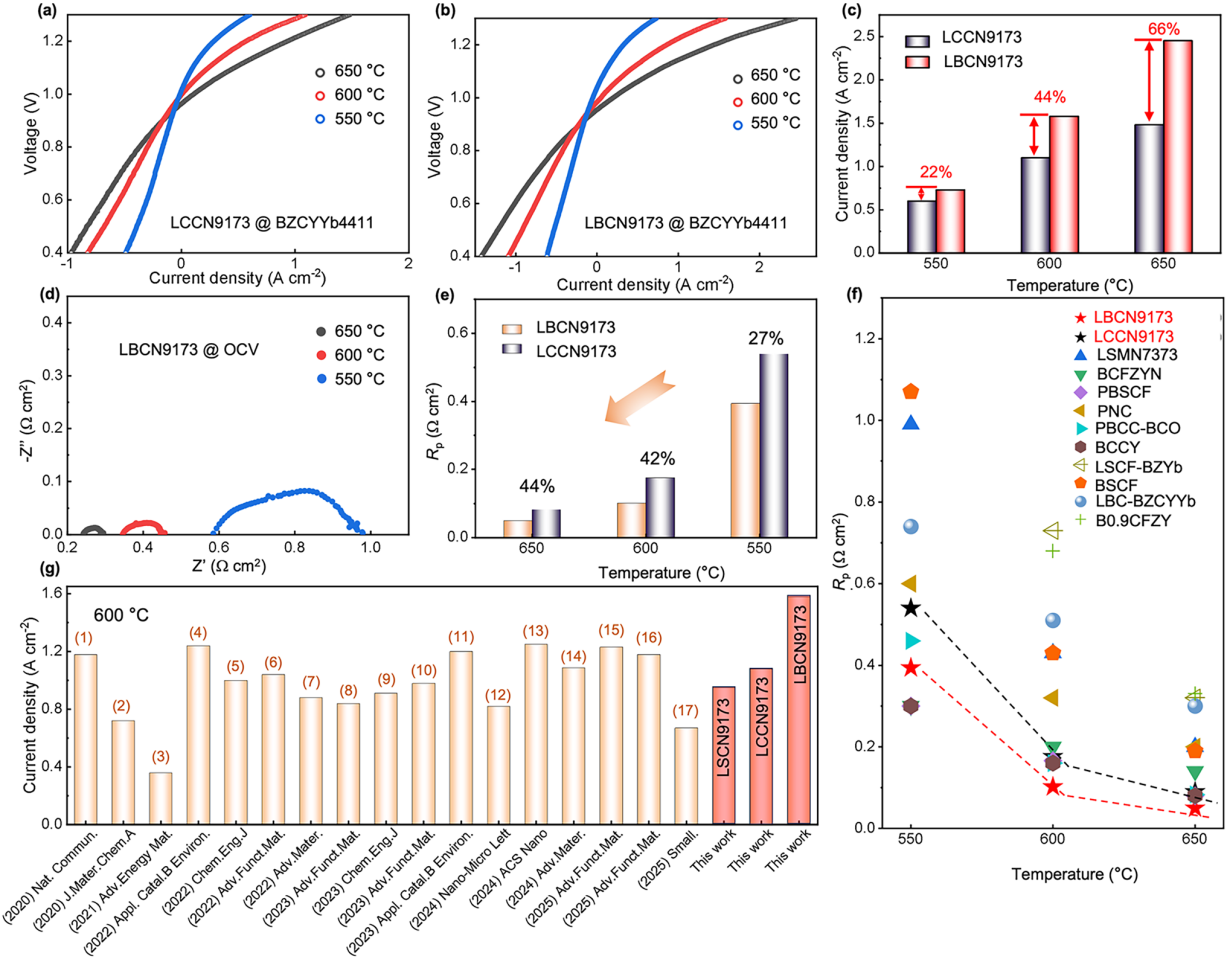
Electrochemical performances of P-SOECs with LBCN9173 and LCCN9173 anodes. **a, b** I-V curves, **c** current densities at 1.3 V, **d** EIS, and **e** *R*_p_ for P-SOECs with LCCN9173 and LBCN9173 anodes. Summary of **f** *R*_p_ and **g** current densities at 1.3 V of this report and other state-of-art anodes. The compositions of oxides in **f**: LSMN7373 = La_0.7_Sr_0.3_Mn_0.7_Ni_0.3_O_3−δ_ [45], BCFZYN = Ba_0.95_(Co_0.4_Fe_0.4_Zr_0.1_Y_0.1_)_0.95_Ni_0.05_O_3−δ_ [56], PBSCF[35], PrNi_0.5_Co_0.5_O_3−δ_(PNC) [57], PBCC-BCO = PrBa_0.8_Ca_0.2_Co_2_O_5+δ_–BaCoO_3−δ_ [58], BCCY = BaCo_0.7_(Ce_0.8_Y_0.2_)_0.3_O_3−δ_ [59], LSCF6482-BZYb = La_0.6_Sr_0.4_Co_0.2_Fe_0.8_O_3−δ_–BaZr_0.8_Yb_0.2_O_3−δ_ [60], LBC-BZCYYb = La_0.6_Ba_0.4_CoO_3−δ_–BaZr_0.1_Ce_0.7_Y_0.1_Yb_0.1_O_3−δ_ [61], B0.9CFZY = Ba_0.9_Co_0.4_Fe_0.4_Zr_0.1_Y_0.1_O_3−δ_ [23]. The oxides in **g** from left to right: (1) PNC [57], (2) (PrBa_0.8_Ca_0.2_)_0.95_Co_2_O_6−δ_ [62], (3) Sr_0.9_Ce_0.1_Fe_0.8_Ni_0.2_O_3−δ_ [63], (4) Ba(Co_0.4_Fe_0.4_Zr_0.1_Y_0.1_)_0.95_Mg_0.05_O_3−δ_ [64], (5) Ba_0.5_Sr_0.5_(Co_0.8_Fe_0.2_)_0.95_P_0.05_O_3−δ_ [65], (6) PrBaCo_1.6_Fe_0.2_Nb_0.2_O_5+δ_ [66], (7) LCCN7382 [29], (8) LCN91 [28], (9) BaCo_0.4_Fe_0.4_Nb_0.1_Sc_0.1_O_3−δ_ [67], (10) Ba_0.5_Sr_0.5_(Co_0.8_Fe_0.2_)_0.9_Er_0.1_O_3−δ_ [68], (11) BCFZYN [56], (12) Sr_2.8_Fe_1.8_Nb_0.2_O_7−δ_ [69], (13) PrSrCo_1.8_Nb_0.2_O_6−δ_ [70], (14) Ba(Co_0.4_Fe_0.4_Zr_0.1_Y_0.1_)_0.95_Ni_0.05_F_0.1_O_2.9−δ_ [71], (15) BSCsCFZr [72], (16) LCCFN‐Cr [14], (17) Ba_0.95_La_0.05_(Fe_0.8_Zn_0.2_)_0.9_Ni_0.1_O_3−δ_ [73].

The electrochemical performances were tested at temperatures ranging from 550 to 650 °C with wet synthetic air (20% Vol. O_2_ and 80% Vol. Ar) supplied into the anode and 3% H_2_O/97% H_2_ fed to the cathode. Figure 7a, b illustrates the *I*–*V* curves of P-SOECs using LBCN9173 and LCCN9173 anodes. P-SOEC with LBCN9173 anode exhibits the current densities of 0.73, 1.58, and 2.45 A cm^−2^ at 1.3 V at 550, 600, and 650 °C, respectively, while those of LCCN9173 cell demonstrated lower values of 0.60, 1.10, and 1.48 A cm^−2^ at 1.3 V. The current densities of LBCN9173 cell are 22%, 44%, and 66% higher than those of LCCN9173 cell at each above-mentioned temperatures (Fig. 7c), attributing to the higher catalytic activity and stronger proton uptake and conduction ability of LBCN9173 oxide as earlier discussed. The P-SOEC with LBCN9173 anode can stably operate for 40 h without obvious degradation at a constant current density of 0.5 A cm^−2^ and 550 °C (Fig. S24). Meanwhile, the *I–V* curves of P-SOEC with LBCN9173 anode were tested by applying the wet gas with hydrogen concentrations ranging from 40 to 80% (Fig. S25) at 600 °C. The current density at 1.3 V of LBCN9173 cell is 1.59, 1.56, and 1.64 A cm^−2^, as hydrogen fraction is 80%, 60%, and 40%, respectively. The data indicates that hydrogen concentration has minimal influence on P-SOEC current density. This is likely because hydrogen, as a product, slightly impedes the HER reaction as its concentration rises.

As the best known to us, other alkaline earth metals, such as Sr, could be considered as A-site dopants and have a possibility of exhibiting high performances. La_0.9_Sr_0.1_Co_0.7_Ni_0.3_O_3−δ_ (LSCN9173) is characterized by the same experimental conditions with LBCN9173 and LCCN9173 (Figs. S26 and S27). LSCN9173 shows a current density of 0.95 A cm^−2^ at 1.3 V and 600 °C, which is inferior to those of LBCN9173 (1.58 A cm^−2^) and LCCN9173 (1.1 A cm^−2^). Additionally, as compared with other oxides containing Sr/Cs/La/Pr/Ce at A-site (Table S6), the performance of LBCN9173 cell still leads among them [57, 62, 70, 72, 73]. Encouragingly, our cell with LBCN9173 (1.58 A cm^−2^) anode is comparable to or better than most of the reported anodes [14, 28, 29, 56, 57, 62–77], such as PNC (1.17 A cm^−2^) [57], BCFZY (1.0 A cm^−2^) [78], Ba_0.95_La_0.05_(Fe_0.8_Zn_0.2_)_0.9_Ni_0.1_O_3−δ_ (BLFZN0.1, 0.67 A cm^−2^) [73], and Ba_0.4_Sr_0.5_Cs_0.1_Co_0.7_Fe_0.2_Zr_0.1_O_3−δ_ (BSCsCFZr, 1.23 A cm^−2^) [72]. These results strongly validate the superiority of Ba as an A-site dopant and further support the potential of LBCN9173 as a high-performance anode material for P-SOEC (Fig. 7g).

Figures 7d and S28 display the typical electrochemical impedance spectra (EIS) of the two cells under open circuit voltage (OCV) condition. Polarization resistance (*R*_p_) and ohmic resistance (*R*_o_) can be extracted from the difference of the low and high frequency intercepts of the EIS with the real axis and the high frequency intercept of the EIS with the real axis. The *R*_p_ of LBCN9173 cell is 0.394, 0.102, and 0.05 Ω cm^2^ lowering 27%, 42%, and 44% than these of LCCN9173 cell at 550, 600, and 650 °C, respectively (Fig. 7e). Figure. 7f presents a comparison of *R*_p_ for this work and the state-of-art anodes [23, 35, 45, 56–61]. LBCN9173 cell exhibits a much lower *R*_p_ (0.102 Ω cm^2^) at 600 °C compared to these anodes, such as BCCY (0.16 Ω cm^2^) [59] and PBSCF (0.16 Ω cm^2^) [35]. These excellent electrochemical performances in terms of current density and *R*_p_ of P-SOEC reinforce that LBCN9173 oxide is a highly effective anode for water electrolysis.

**Fig. 8:**
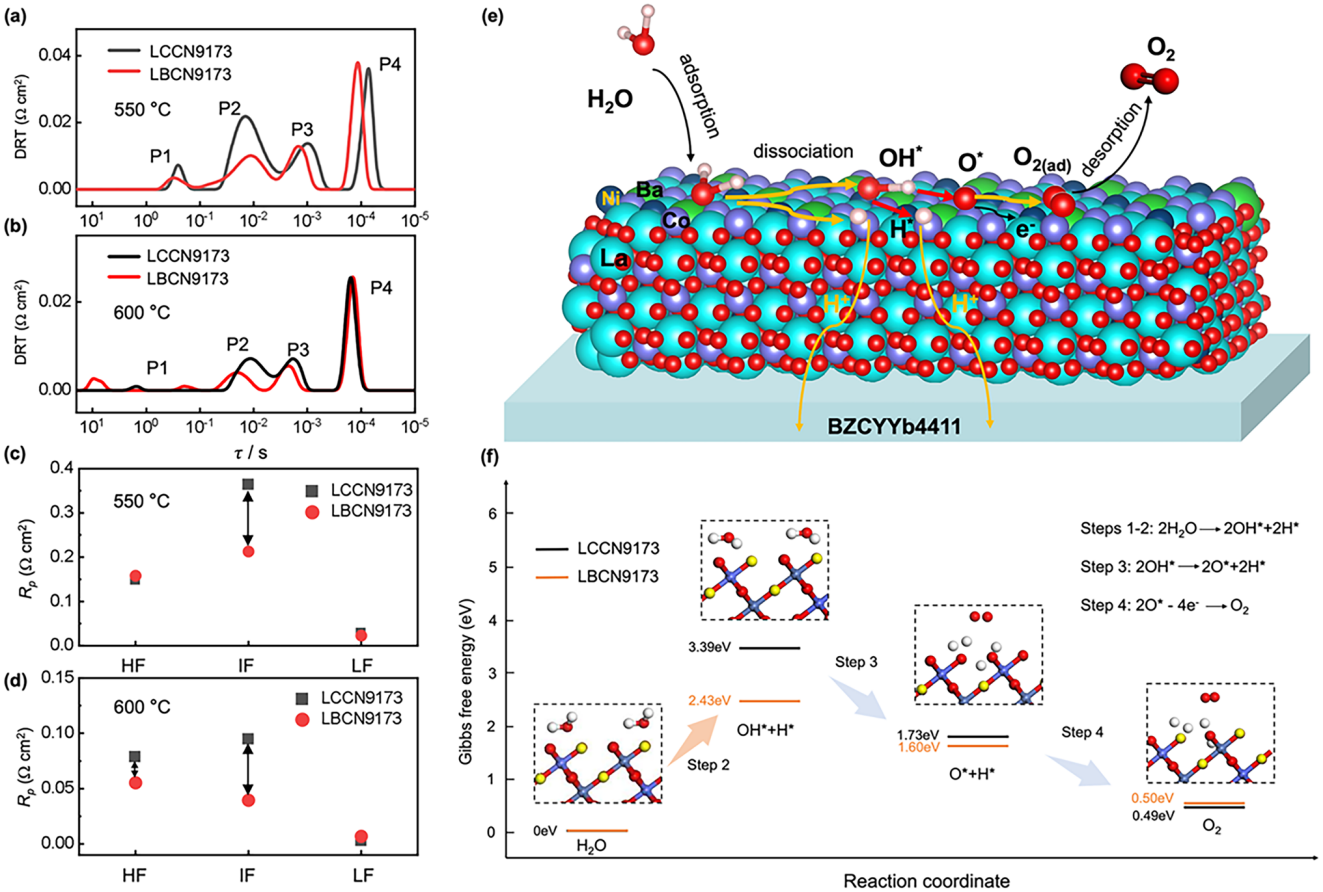
**a, b** DRT plots and **c, d** *R*_HF_, *R*_IF_, and *R*_LF_ values for LBCN9173 and LCCN9173 cells under OCV condition at 550 and 600 °C. **e** Schematic WOR mechanism and **f** Gibbs free energy of main WOR steps on (001) surface of LBCN9173 anode

DRT analysis is an effective tool for decomposing complex and overlapped electrochemical processes into distinct or single processes by identifying the characteristic distribution of typical EIS timescales. In order to gain deeper insights into the water electrolysis reaction processes at the anode of the two cells, we employed DRT analysis on EIS measured at 550 and 600 °C. As illustrated in Fig. 8a, b, the electrochemical processes were deconvolved into 4 peaks, labeled P1, P2, P3, and P4. Among the four peaks, P1 corresponding to *R*_LF_ is related to O_2_ desorption (step 5 in Table S7). P2 and P3 relevant to *R*_IF_ correspond to the H_2_O adsorption/dissociation and O_2_ formation processes (steps 1–4 in Table S7). P4 is associated with the charge transfer process, mirroring *R*_HF_ (step 6 in Table S7) (where LF, IF, and HF mean low frequency, intermediate frequency, and high frequency, respectively) [28]. The main difference in resistance between LBCN9173 and LCCN9173 cells lies in the IF resistances (Fig. 8c, d). *R*_IF_ of LBCN9173 cell with the values of 0.213 and 0.039 Ω cm^2^ is lower than that of LCCN9173 cell (0.364 and 0.094 Ω cm^2^) at 550 and 600 °C (Table S7), respectively, indicating faster reaction kinetics at LBCN9173 anode.

Water oxidation reaction (WOR) mainly relates to steps1-4, shown in Figs. 8e and S29a, and Table S7, whose Gibbs energies were calculated on the (001) surface of LBCN9173 and LCCN9173 models with 120 atoms (Figs. 8f and S29b-e). The results show that the rate-determined step of WOR for the two cells is the dissociation of H_2_O (steps 1–2) (Fig. 8f). The calculating results align well with the DRT analysis (*R*_IF_ is much higher than R_LF_ shown in Fig. 8c, d and Table S7). The WOR energy barrier of LBCN9173 cell (3.39 eV) is lower than that of LCCN9173 cell (2.43 eV), which provides crucial insights into the superior electrochemical performances of LBCN9173 cell compared to LCCN9173 cell.

The EIS spectra of LBCN9173 and LCCN9173 cells under different oxygen partial pressures (*p*_O2_ = 10%, 20%, 40%) at 600 °C were analyzed for further exploring the anodic reactions of P-SOEC. Shown in Fig. S30a, b, the *R*_p_ decreases from 0.161 to 0.086 Ω cm^2^ (LBCN9173 cell) and 0.209 to 0.127 Ω cm^2^ (LCCN9173 cell) as *p*_O2_ increased from 10 to 40%. The DRT analysis results suggest that the R_IF_ and R_HF_ show the same trend as the *R*_p_. According to the function equation of *R* = *k*(*p*_O2_)^−*n*^, the correlation coefficient (*n*) between resistance at different frequencies is calculated, as shown in Fig. S30c, d. The *n* values for both LBCN9173 (0.54 *vs* 0.37) and LCCN9173 (0.48 *vs* 0.25) cells in the IF range are greater than those in the HF range, indicating that the *p*_O2_ primarily affects the *R*_IF_ during the anodic reaction processes. Combined with the above DFT calculation result that *R*_IF_ is the critical reaction steps at the anode of the two P-SOECs, it is easy to infer that the ${\text{V}}_{\text{O}}^{\cdot\cdot}$ concentration has more effects on *R*_p_. LBCN9173 oxide shows higher ${\text{V}}_{\text{O}}^{\cdot\cdot}$ concentration, and thus the lower* R*_p_ and the higher current densities (Figs. S8, S9 and Table S2). In conclusion, LBCN9173 oxide proves to be a high-performance anode for P-SOECs due to the synergistic enhancement of proton conductivity and catalytic activity. As illustrated in Fig. S31a, b**,** when the anode without or with low proton conductivity is used as the P-SOEC anode, the reaction active areas are confined to the triple-phase boundary (TPB) or result in ion accumulation at the reaction active sites, and thus the bad performance [59]. If the anode with high proton conductivity suffers in low catalytic activity, which will limit the generation of sufficient protons to the anode, thereby restricting the improvement of current density (Fig. S31c). Therefore, to achieve superior electrochemical performance, both rapid proton conductivity and efficient anode catalytic activity are indispensable [79]. As shown in Fig. S31d, the synergy between catalytic activity and proton conduction significantly enhances the overall performance of the P-SOEC. Overall, these results strongly suggest that the hydration ability of oxides can serve as an excellent indicator to screen the high-performance anode for P-SOECs by the ML model. Looking ahead, it is highly desired to develop a more sophisticated ML framework capable of simultaneously optimizing multiple key properties, particularly catalytic activity and proton conductivity. This advanced model will be trained on comprehensive datasets that incorporate diverse material characteristics, including oxygen vacancy concentration, ASR, proton conductivity, TEC, HPC, hydration enthalpy, etc. [25, 32, 80–82], enabling the identification of optimal oxide compositions and structures that effectively balance these required properties, and thus designing the high-performance anodes with more accuracy. This integrated approach will facilitate efficient exploration of complex oxide systems and accelerate the discovery of next-generation P-SOEC materials with unprecedented performances.

## Conclusion

In summary, La_0.9_Ba_0.1_Co_0.7_Ni_0.3_O_3–*δ*_ (LBCN9173) and La_0.9_Ca_0.1_Co_0.7_Ni_0.3_O_3–*δ*_ (LCCN9173) oxides were successfully synthesized and characterized, which were designed with the assistance of a well-constructed machine learning (ML) model. The experimental hydrated proton concentration (HPCs) of the two oxides matches well with the predicted values, manifesting a high reliability of the ML model. The HPC, hydration enthalpy, and hydration energy of LBCN9173 oxide are superior to those of LCCN9173 oxide. The high HPC (0.052 mol unit^−1^ at 500 °C), negative hydration energy (− 2.3 eV), and low proton conduction energy barrier (0.49 eV) enable the excellent proton conduction of LBCN9173 oxide. The lower free energies for oxygen evolution reaction (OER) processes of LBCN9173 oxide indicate the higher OER catalytic activity due to the higher oxygen vacancy concentration. As a result, P-SOEC with LBCN9173 anode exhibits the current densities of 1.58 and 2.45 A cm^−2^ at 1.3 V at 600 and 650 °C, respectively, higher than that of LCCN9173 cell (1.10 and 1.48 A cm^−2^), which are superior to most of the reported P-SOECs. Overall, LBCN9173 oxide designed by the well-constructed ML model can serve as a promising anode for P-SOEC due to its outstanding proton conduction ability and high catalytic activity.

## Supplementary Information

Below is the link to the electronic supplementary material.

Supplementary file1 (DOCX 12535 KB)
