# Supplementary material for: Machine Learning Tailored Anodes for Efficient Hydrogen Energy Generation in Proton-Conducting Solid Oxide Electrolysis Cells
Source: Nanomicro Lett. 2025 May 23;17:274. doi: 10.1007/s40820-025-01764-7 (PMC12102459; doi:10.1007/s40820-025-01764-7)
Supplement: Supplementary file 1 — Supplementary file1 (DOCX 12535 KB) [file 40820_2025_1764_MOESM1_ESM.docx]

Supporting Information for

**Machine Learning Tailored Anodes for Efficient Hydrogen Energy Generation in Proton-Conducting Solid Oxide Electrolysis Cells**

Fangyuan Zheng^1#^, Baoyin Yuan^2#^, Youfeng Cai^1^, Huanxin Xiang^1^, Chunmei Tang^1^*, Ling Meng^1^, Lei Du^1^, Xiting Zhang^1^, Yoshitaka Aoki^3^, Ning Wang^1^*, Siyu Ye^1^*

^1^ Huangpu Hydrogen Energy Innovation Center, School of Chemistry and Chemical Engineering, Guangzhou University, Guangzhou 510006, P. R. China

^2^ School of Mathematics and Information Science, Guangzhou University, Guangzhou 510006, P. R. China

^3^ Faculty of Engineering, Hokkaido University, N13W8, Kita-ku, Sapporo, 060-8628, Japan

^#^ Fangyuan Zheng and Baoyin Yuan contributed equally to this work.

*Corresponding authors. E-mail: [siyu.ye@gzhu.edu.cn](mailto:siyu.ye@gzhu.edu.cn) (Siyu Ye); [ningwang@gzhu.edu.cn](mailto:ningwang@gzhu.edu.cn) (Ning Wang); [tangchunmei554@gzhu.edu.cn](mailto:tangchunmei554@gzhu.edu.cn) (Chunmei Tang)

**Supplementary Figures and Tables**


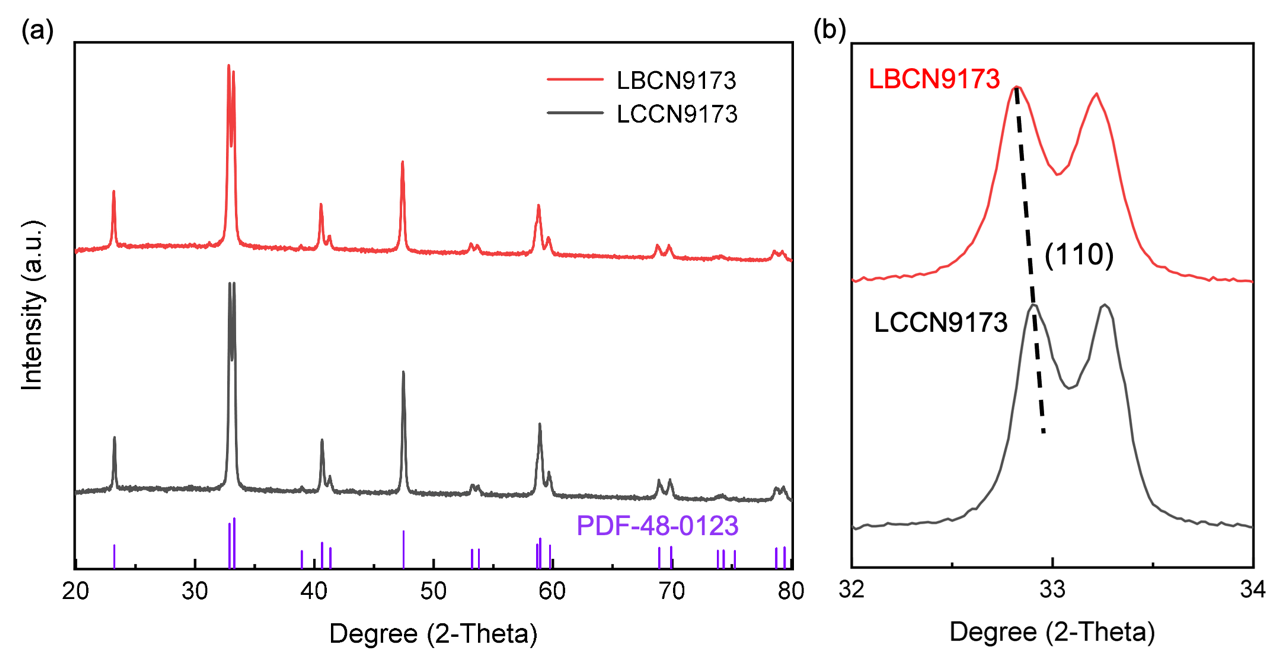


**Fig. S1** (**a**) X-ray diffraction patterns and (**b**) the enlarged (110) peak for LBCN9173 and LCCN9173 oxides


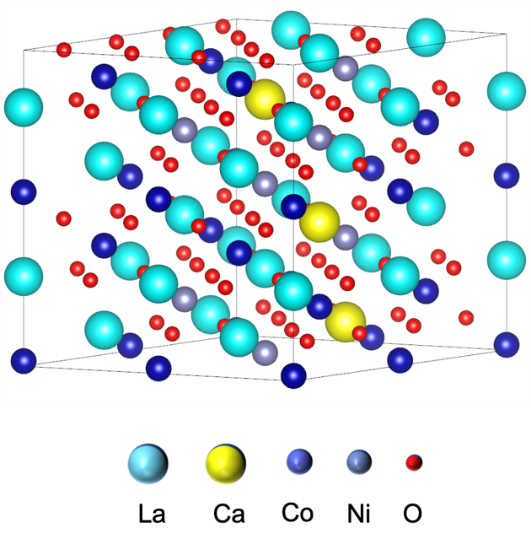


**Fig. S2** Phase structure of LCCN9173 oxide


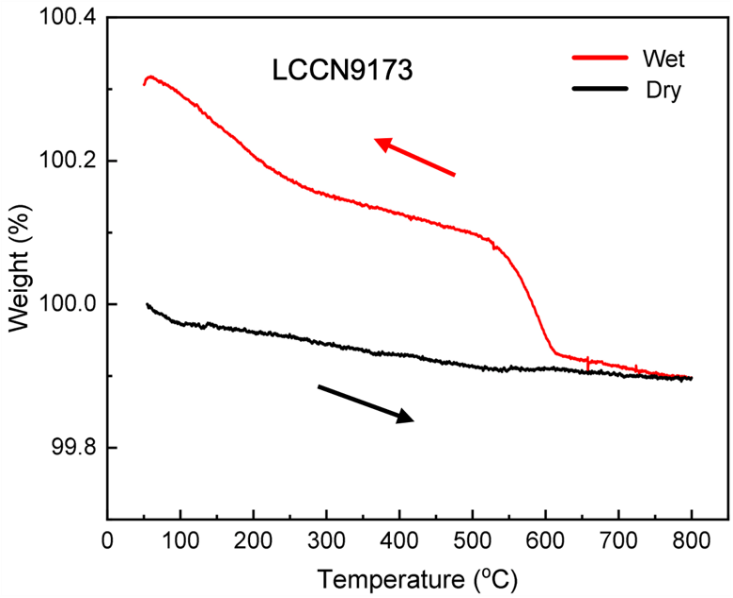


**Fig. S3** TG curves of LCCN9173 oxide under dry and wet air (*p*_H2O_ =0.02 atm) at 50-800 °C


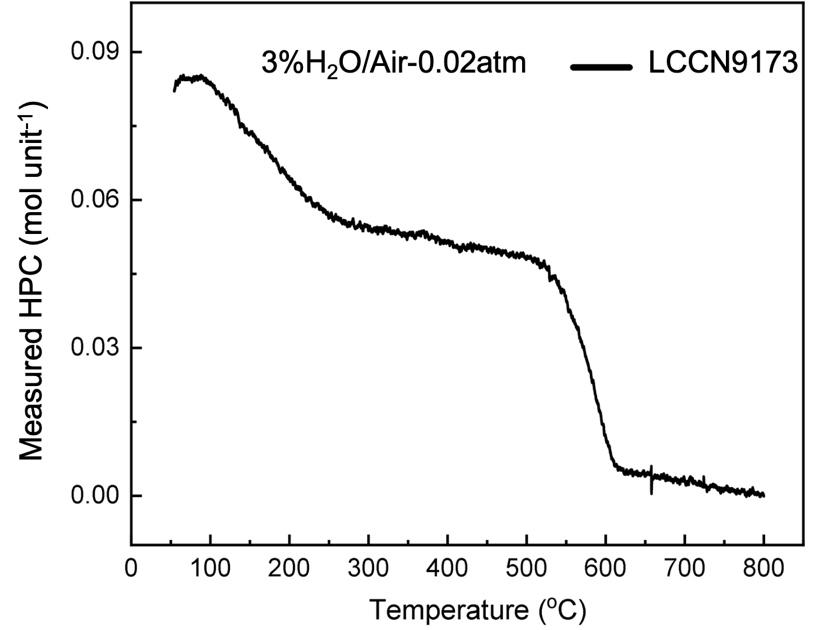


**Fig. S4** Measured hydrated proton concentration (HPCs) of LCCN9173 oxide at 50-800 °C


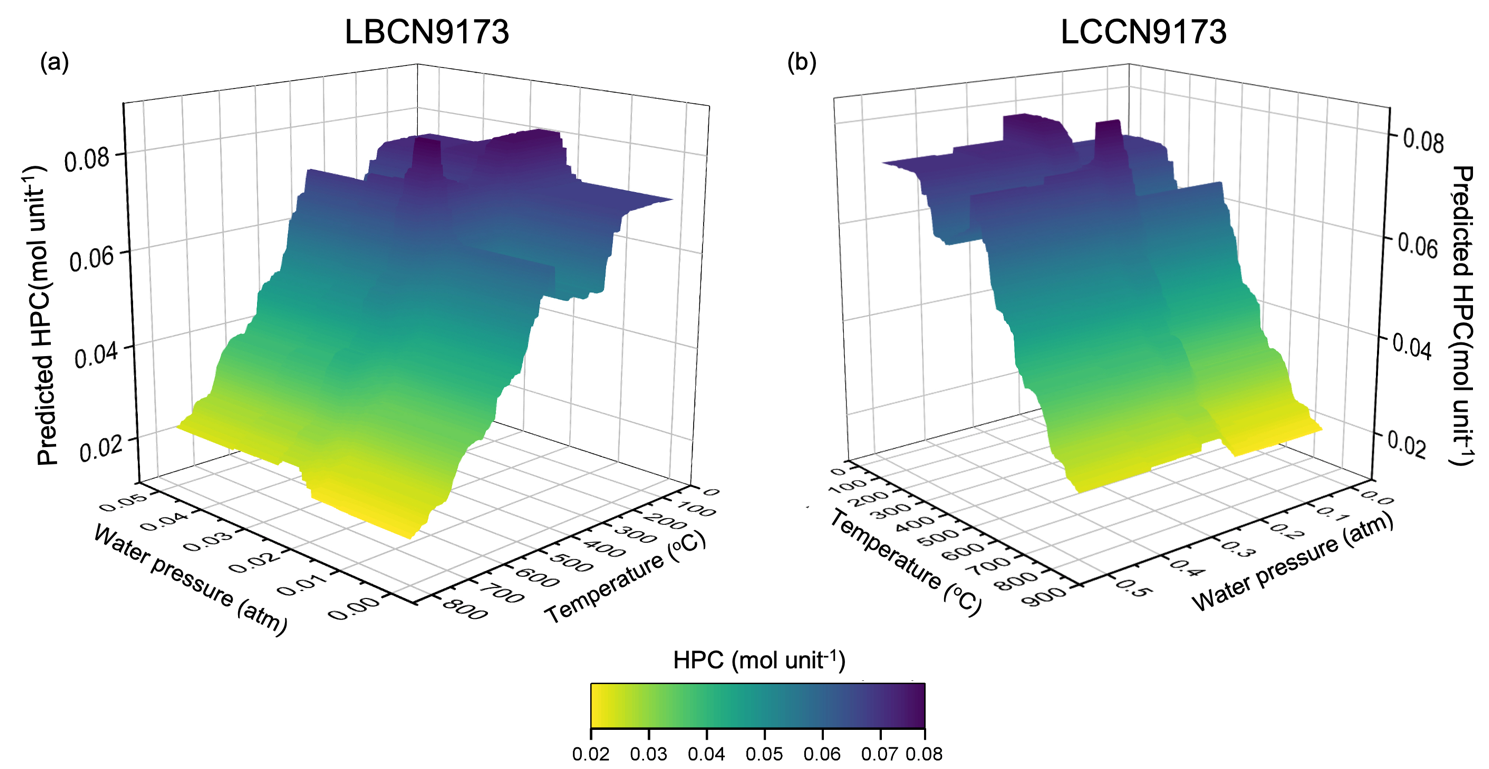


**Fig. S5** Predicted HPCs as functions of *p*_H2O_ (0-0.05 atm) and temperatures (50-800 °C)


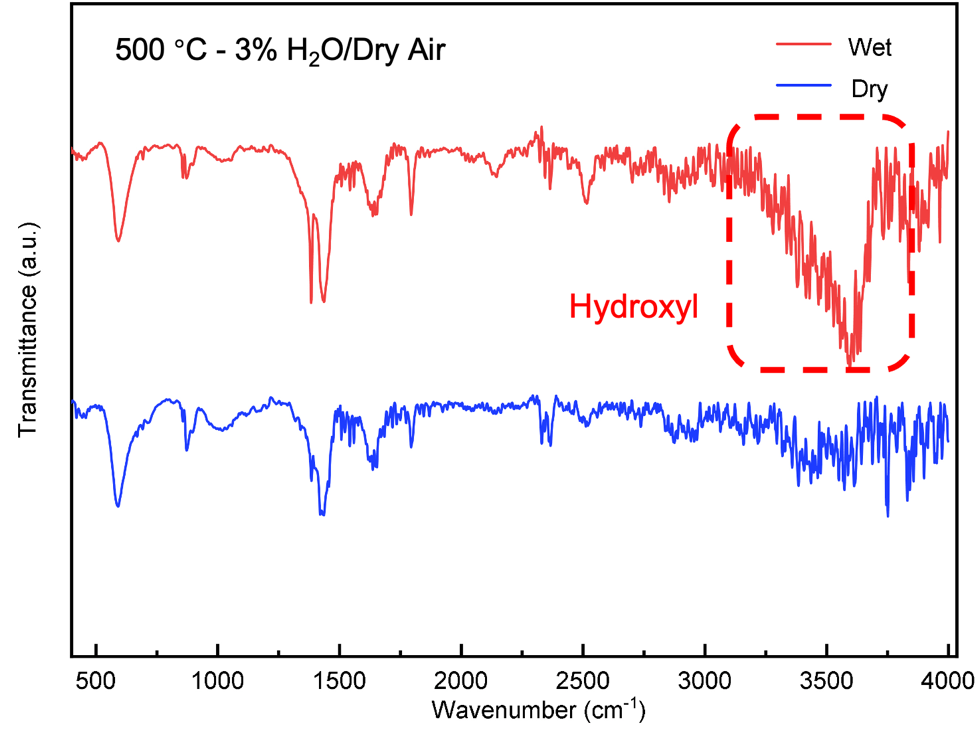


**Fig. S6** Fourier Transform Infrared Spectrometer (FT-IR) profiles for hydrated and dehydrated LBCN9173 samples


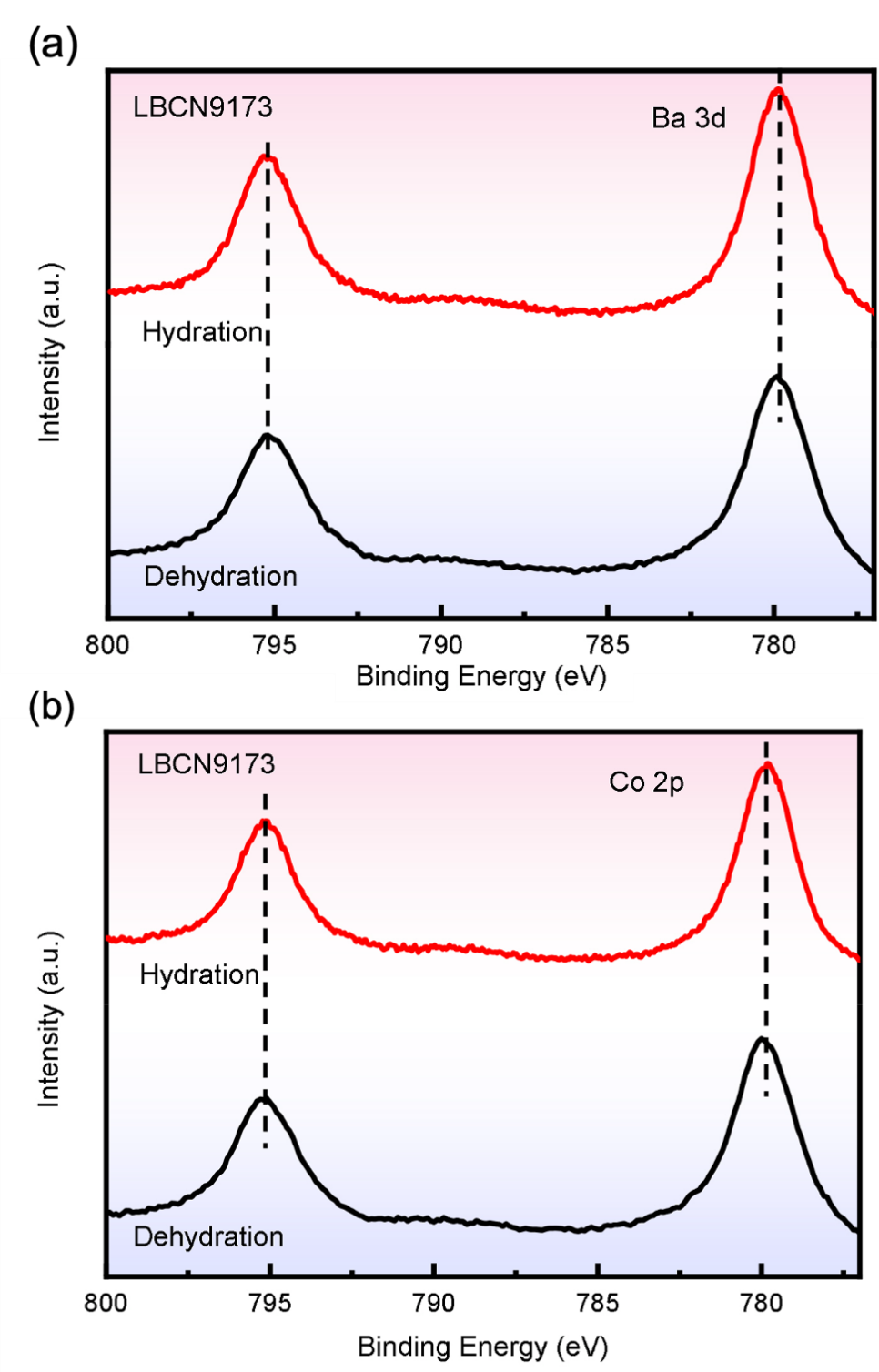


**Fig. S7** X-ray Photoelectron Spectroscopy (XPS) spectra of (**a**) Ba 3d and (**b**) Co 2p for hydrated and dehydrated LBCN9173 samples


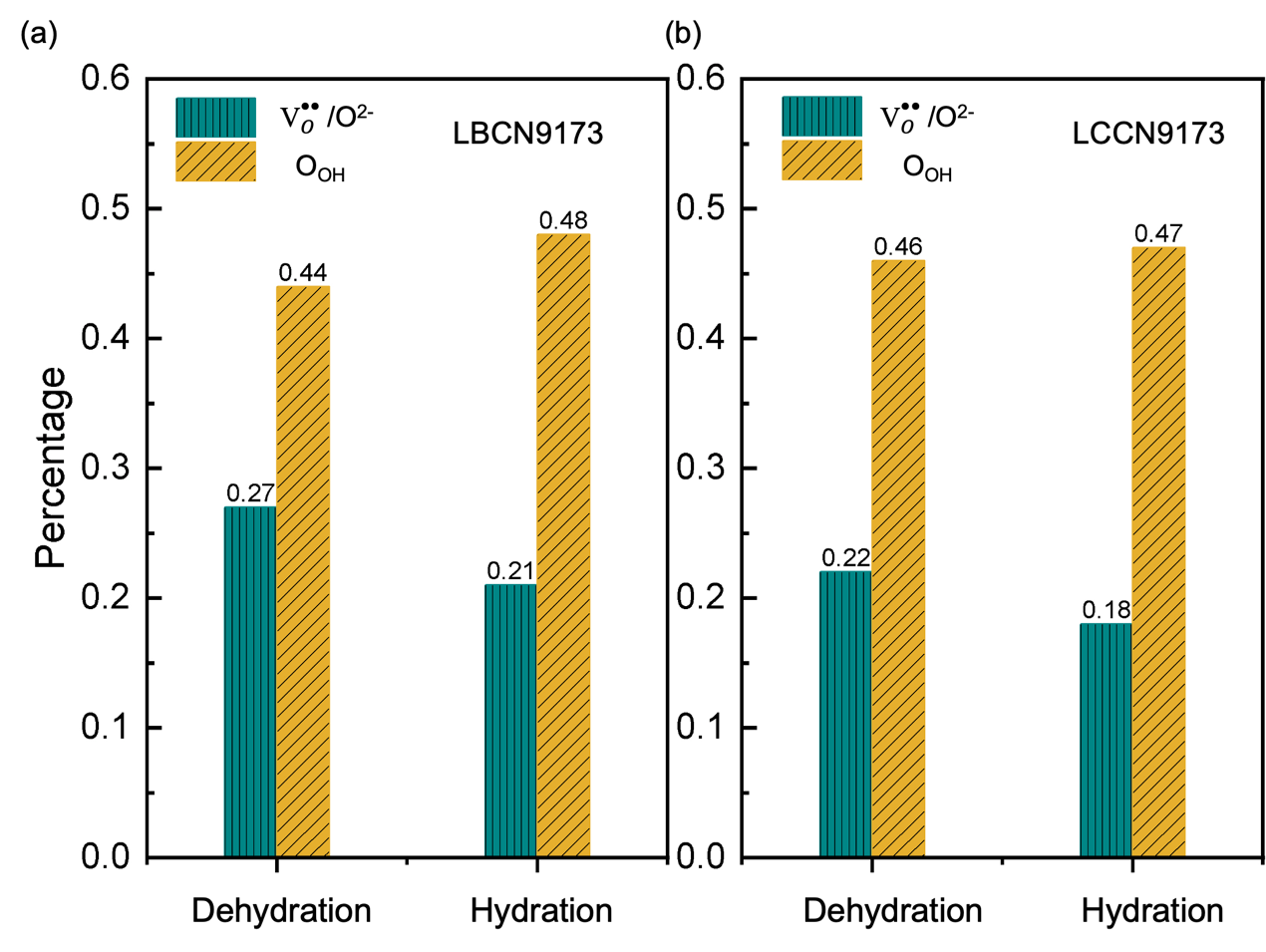


**Fig. S8** Percentage of the $\text{V}_{\text{O}}^{\text{••}}$/O^2-^ and O_OH_ contents for hydrated and dehydrated (**a**) LBCN9173 and (**b**) LCCN9173 oxides


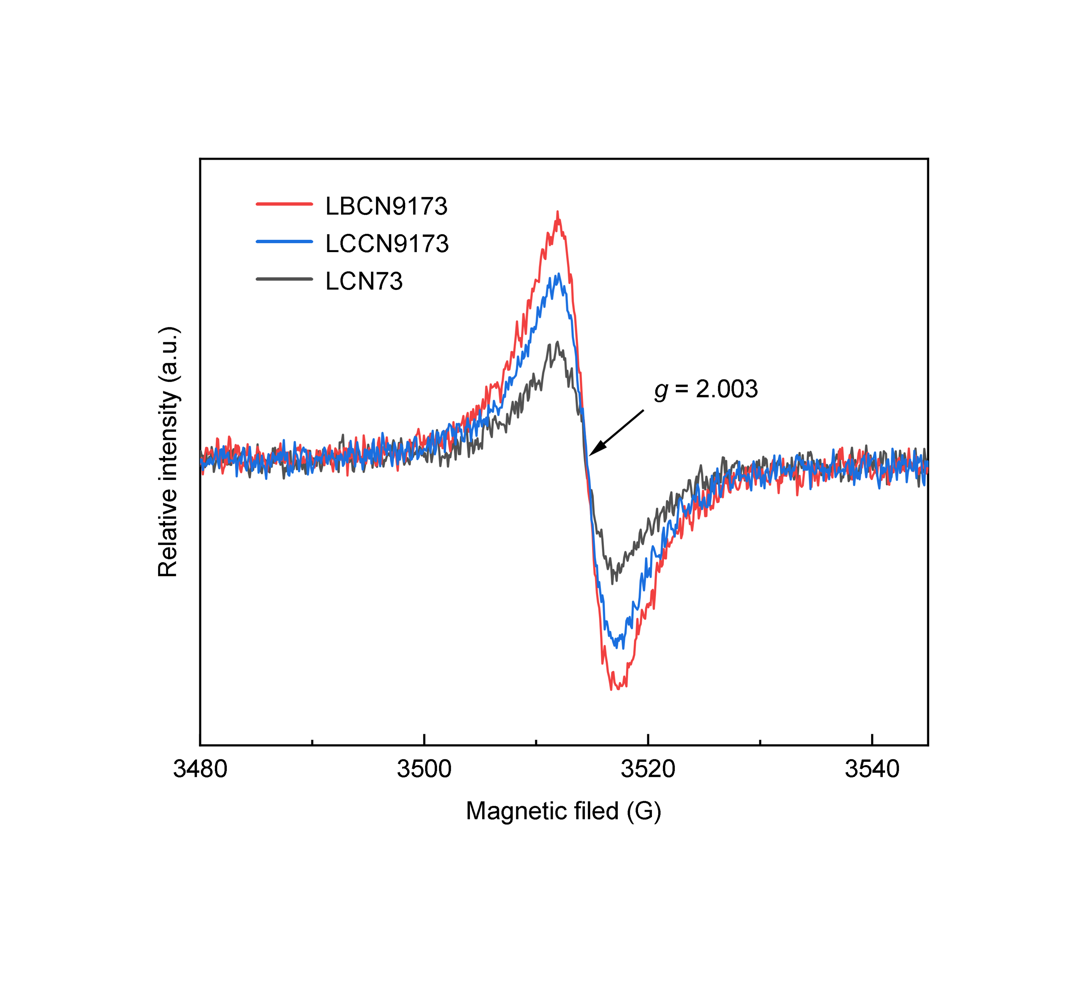


**Fig. S9** EPR spectra of LCN73, LBCN9173, and LCCN9173 oxides


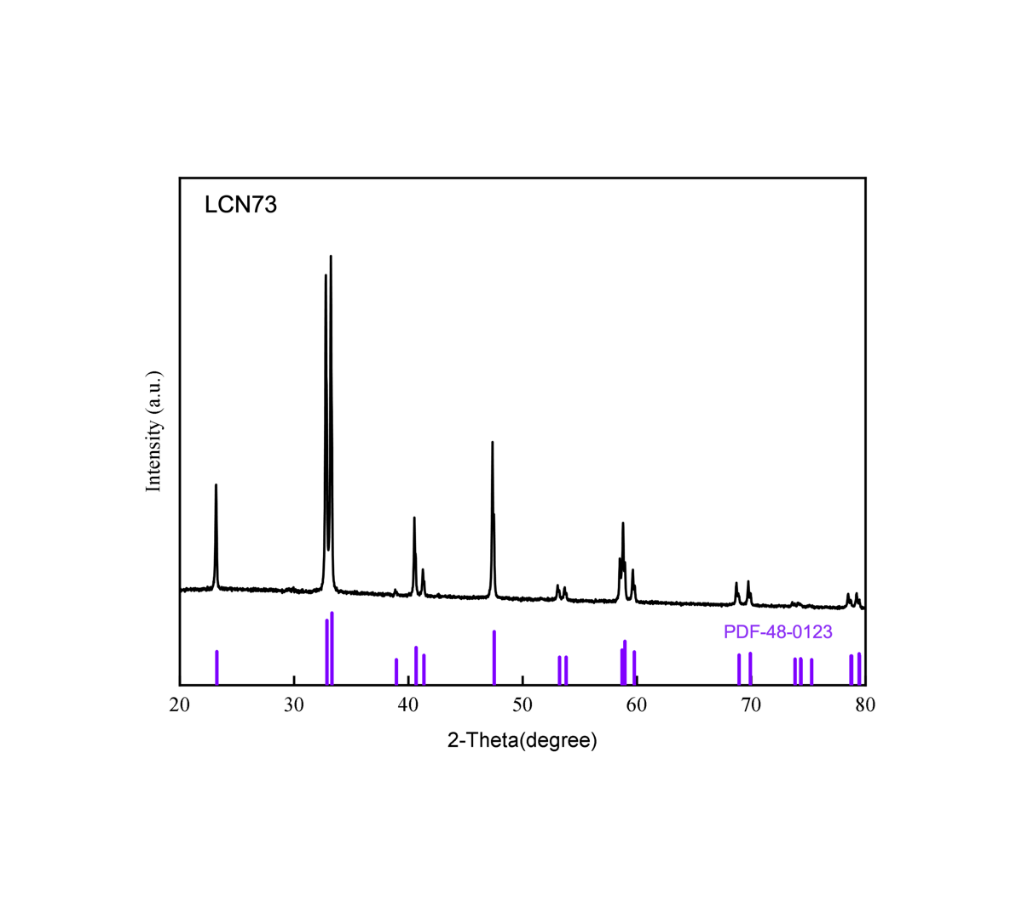


**Fig. S10** XRD pattern of LCN73 oxide


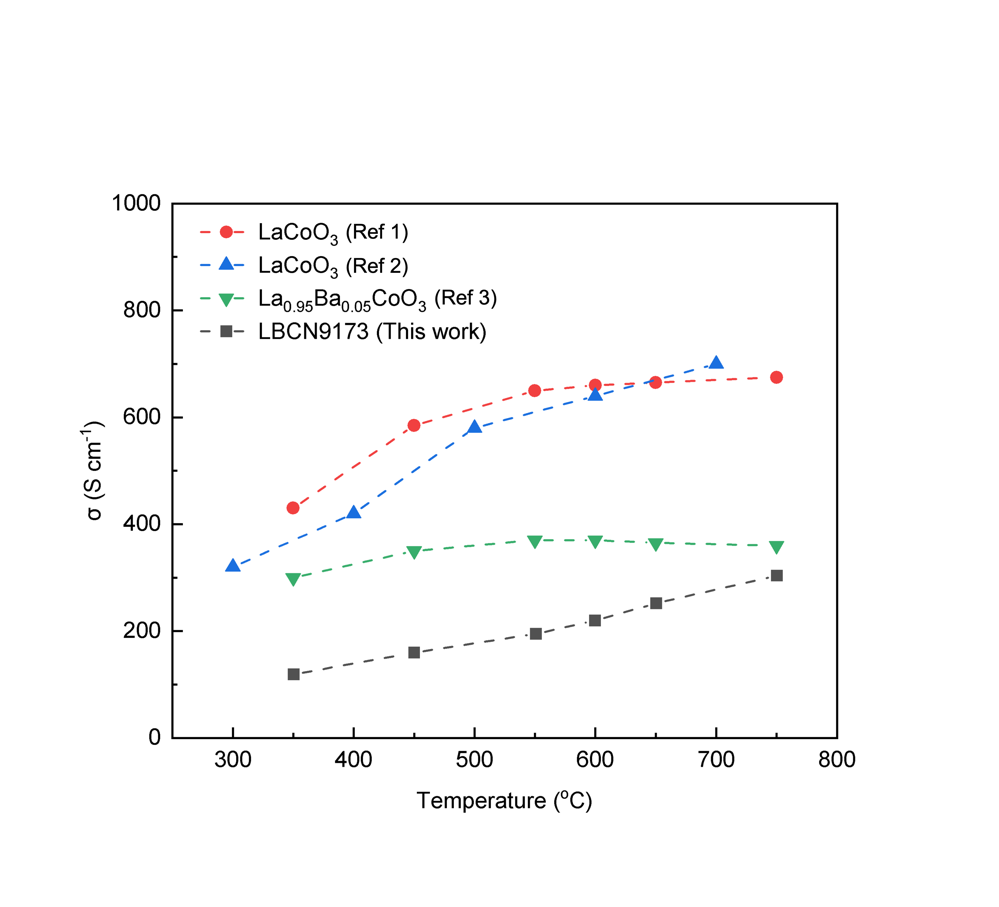


**Fig. S11** Electrical conductivity of LBCN9173 and other reported oxides (LaCoO_3_ and La_0.95_Ba_0.05_CoO_3_) [S1-S3]


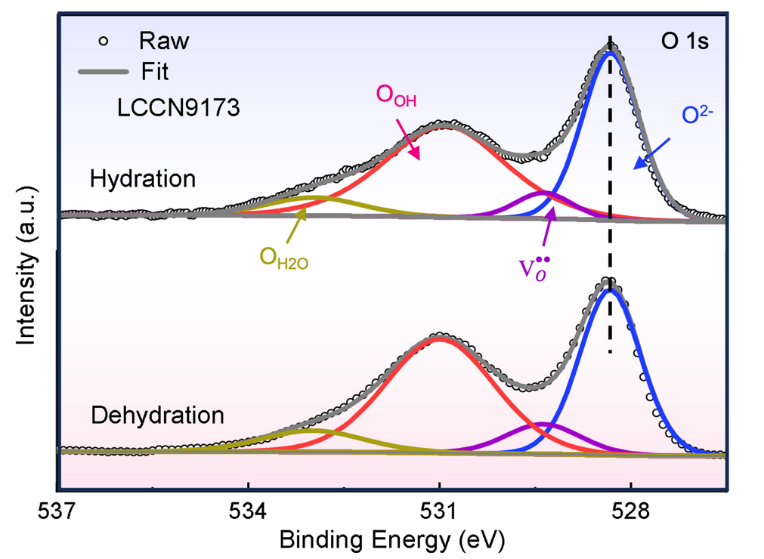


**Fig. S12** XPS spectra of O 1s for hydrated and dehydrated LCCN9173 samples


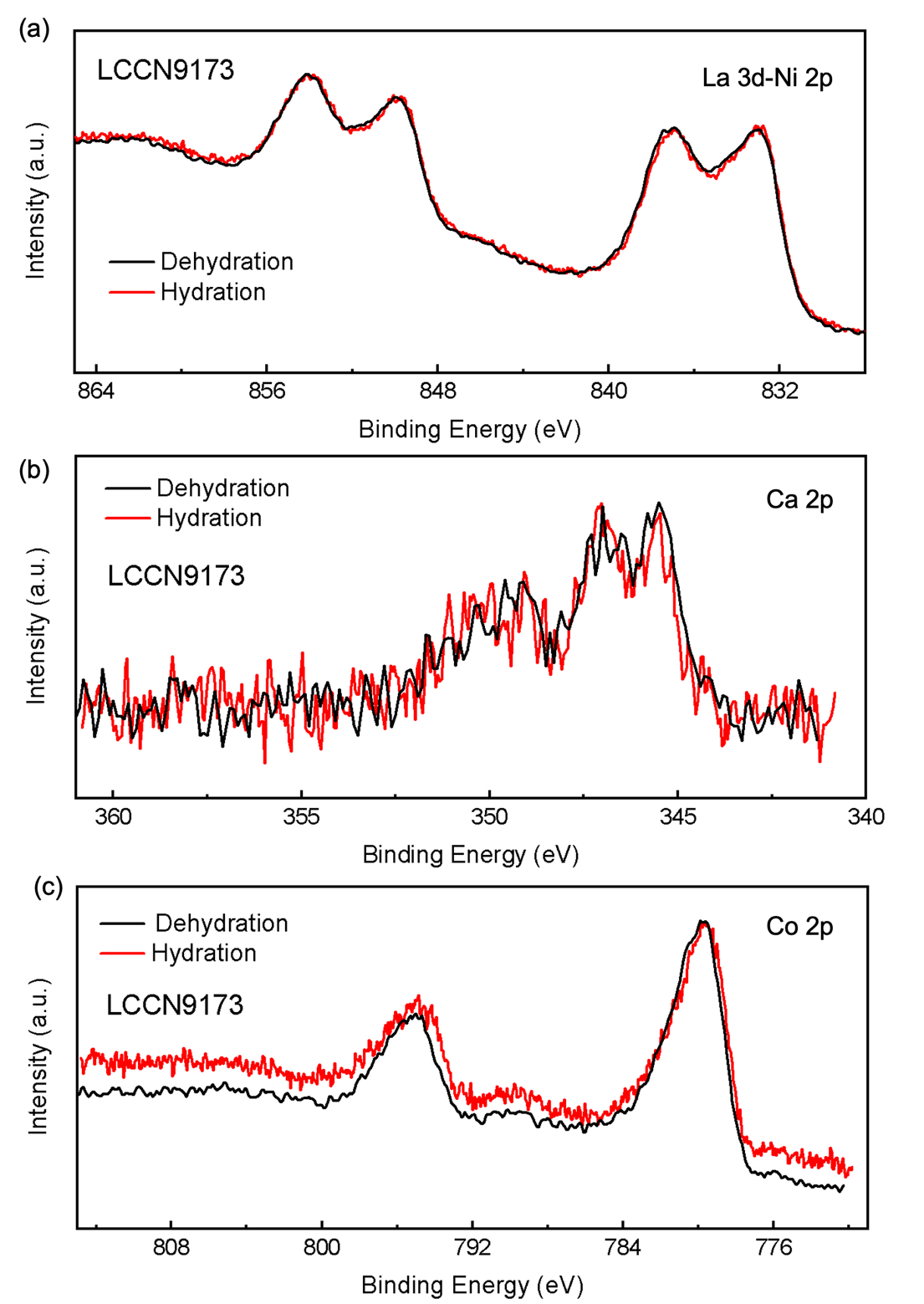


**Fig. S13** XPS spectra of (**a**) La 3d-Ni 2p, (**b**) Ca 2p, (**c**) Co 2p for hydrated and dehydrated LCCN9173 samples


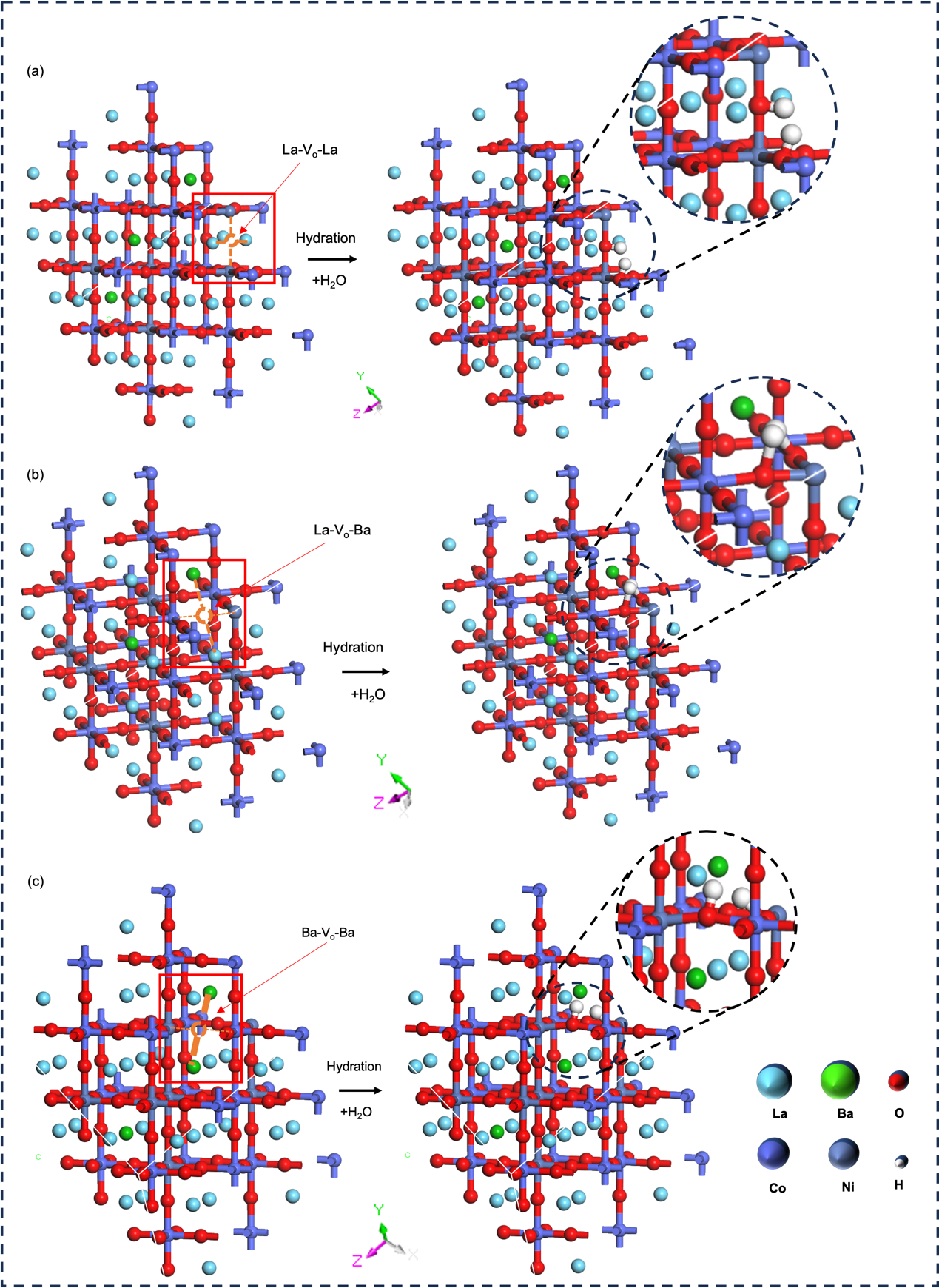


**Fig. S14** Illustration of hydration reaction for LBCN9173 oxides with $\text{V}_{\text{O}}^{\text{••}}$ located between (**a**) La-La, (**b**) La-Ba, (**c**) Ba-Ba cations


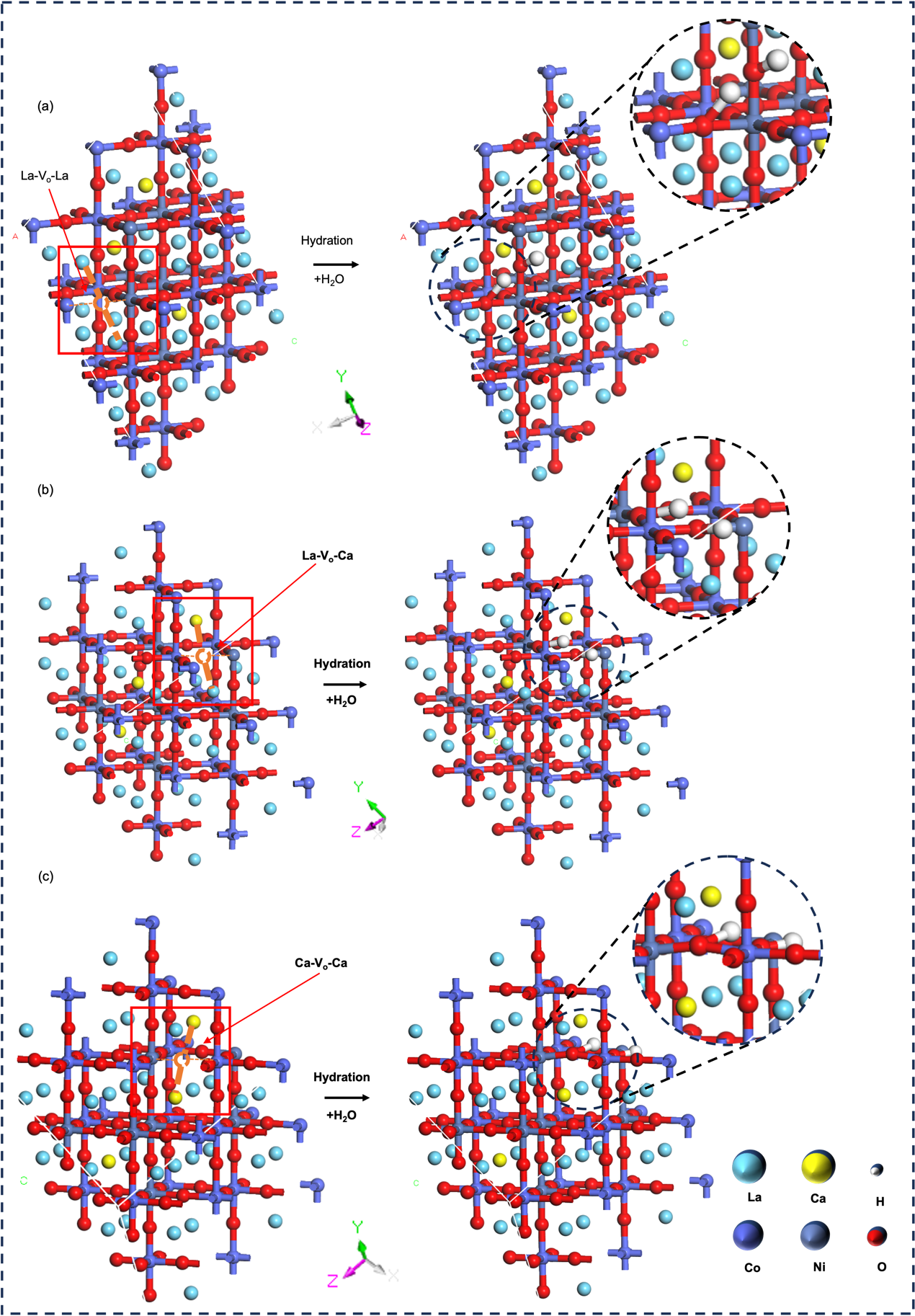


**Fig. S15** Illustration of hydration reaction for LCCN9173 with $\text{V}_{\text{O}}^{\text{••}}$ located between (**a**) La-La, (**b**) La-Ca, (**c**) Ca-Ca cations


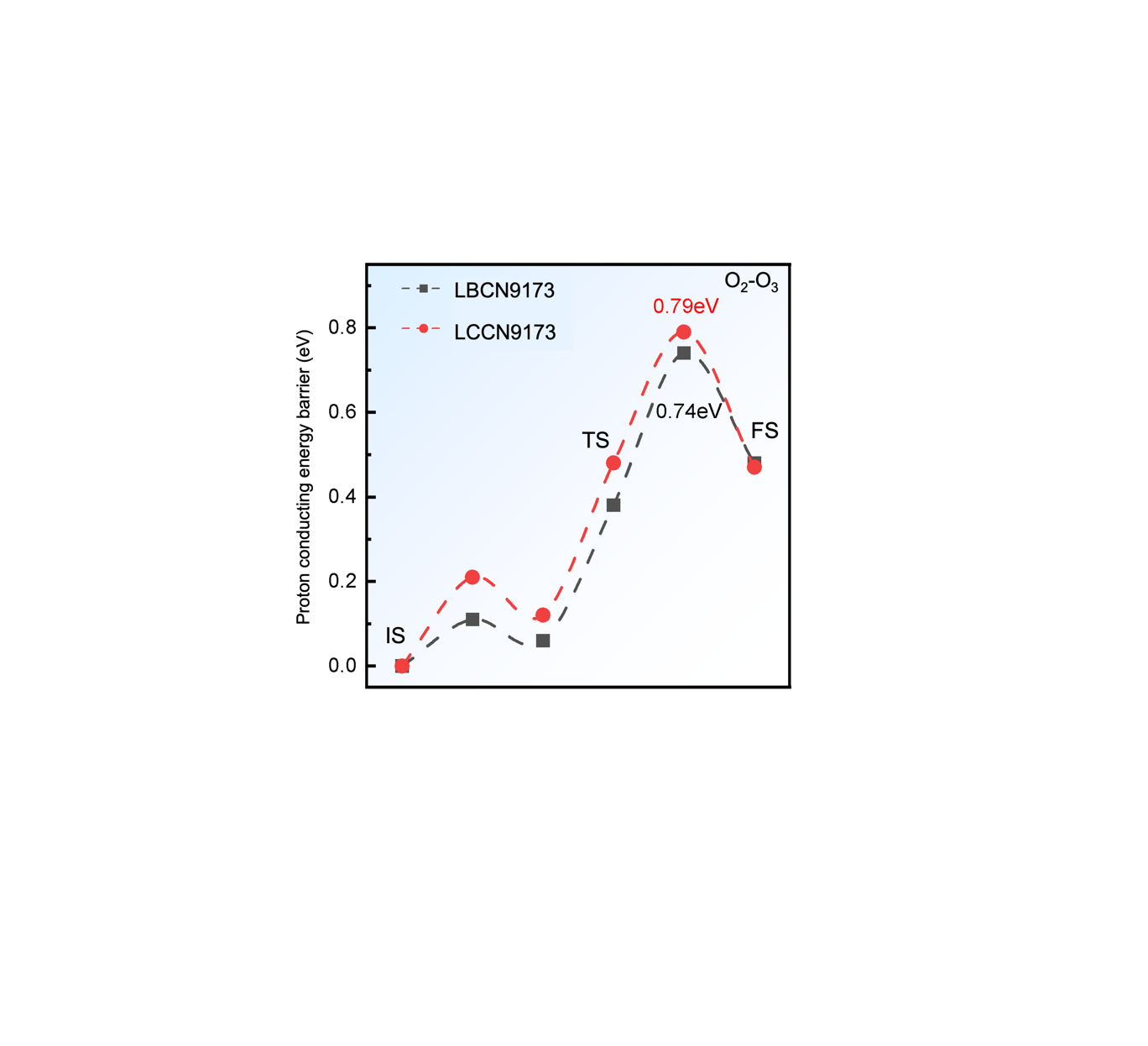


**Fig. S16** Proton conducting energy barriers from O2 to O3 site in LBCN9173 and LCCN9173 oxides


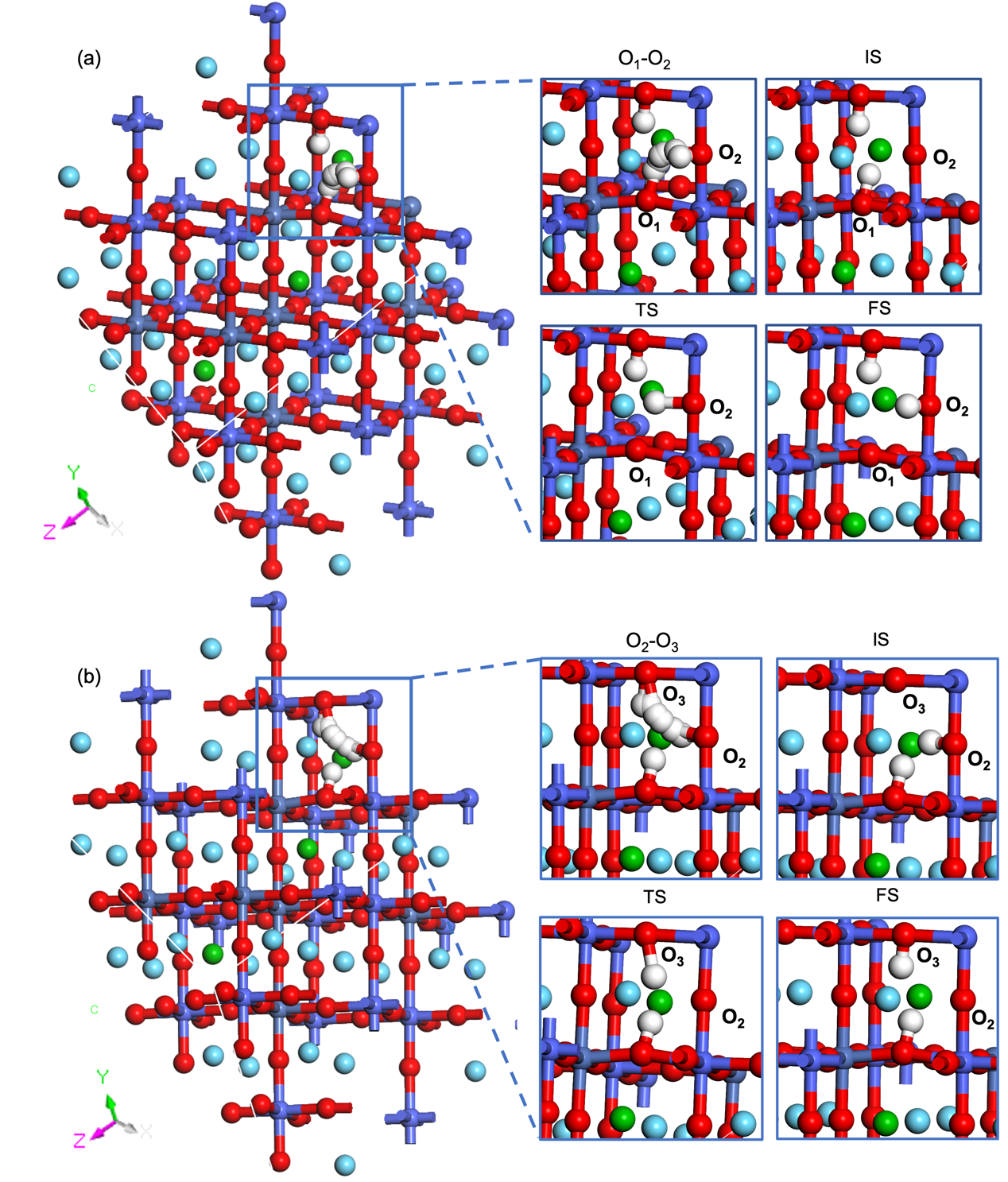


**Fig. S17** Proton conducting path for LBCN9173 oxide during proton transferring from (**a**) O1 to O2 and (**b**) O2 to O3 site


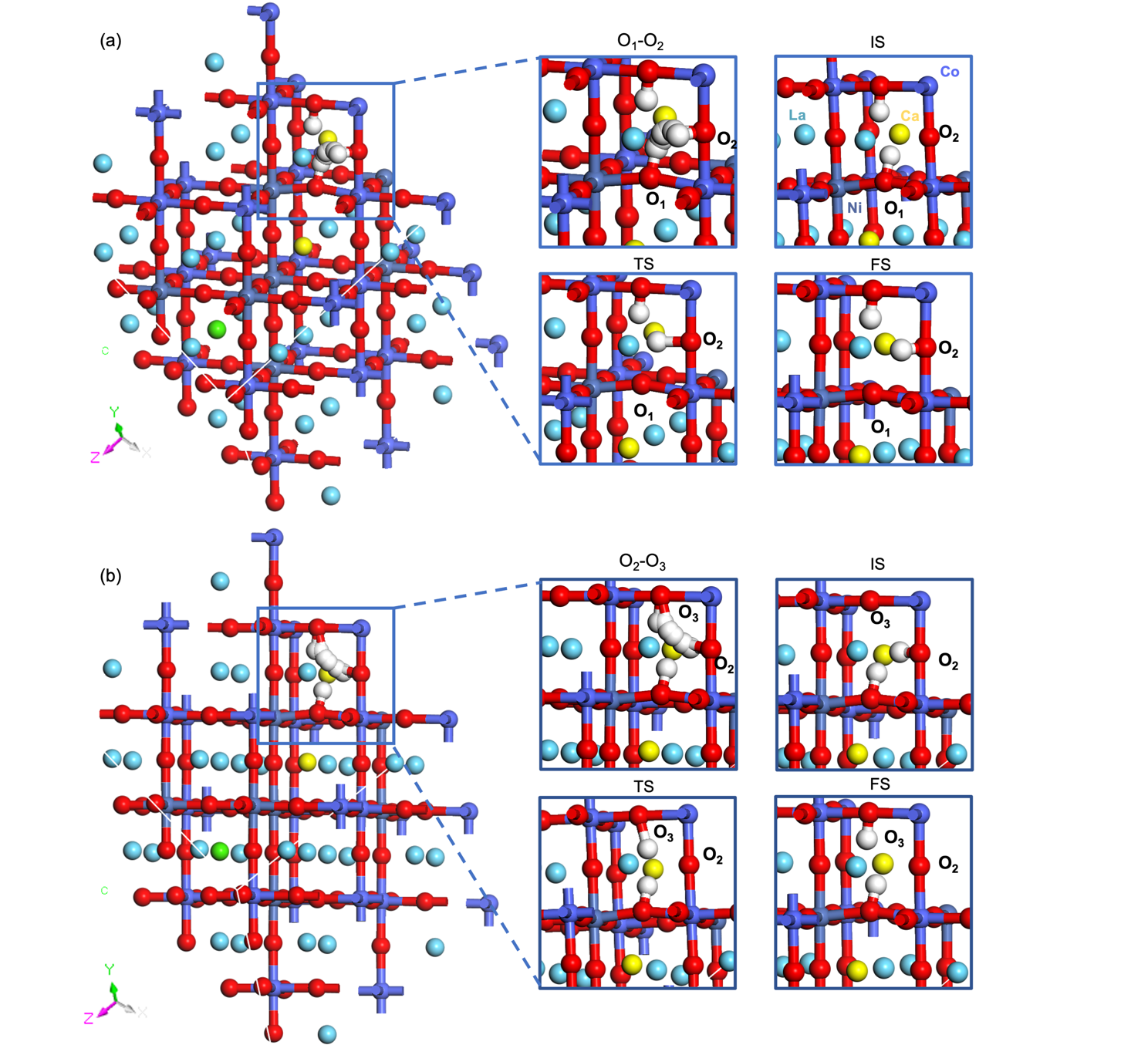


**Fig. S18** Proton conducting path for LCCN9173 oxide during proton transferring from (**a**) O1 to O2 and (**b**) O2 to O3 site


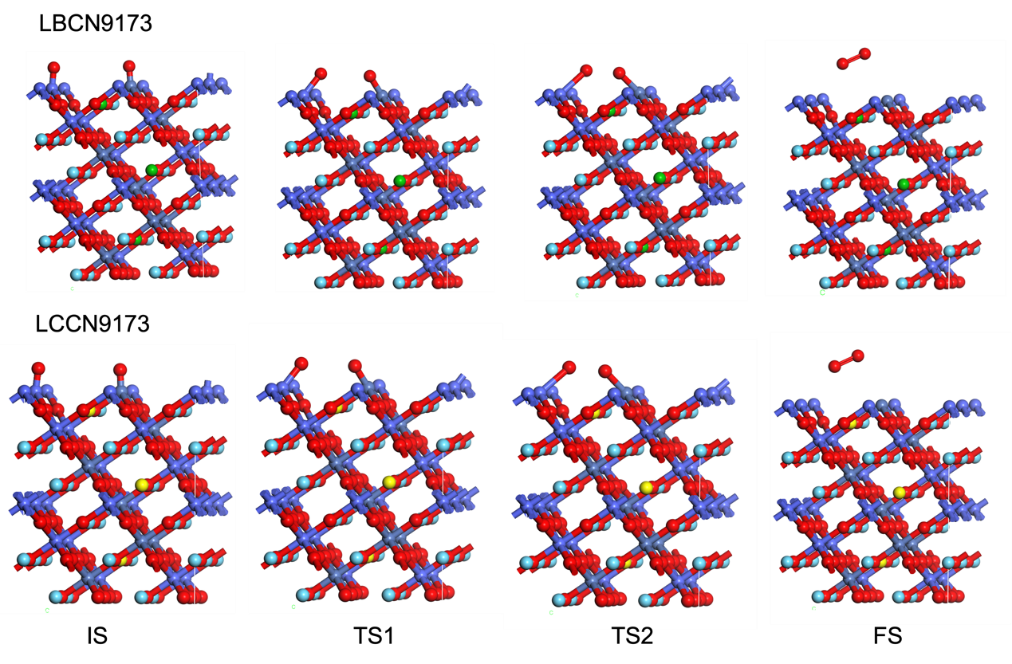


**Fig. S19** OER reaction states (initial state (IS), transition state (TS), and final state (FS)) at Co-Ni sites on (001) surface of LBCN9173 and LCCN9173 oxides


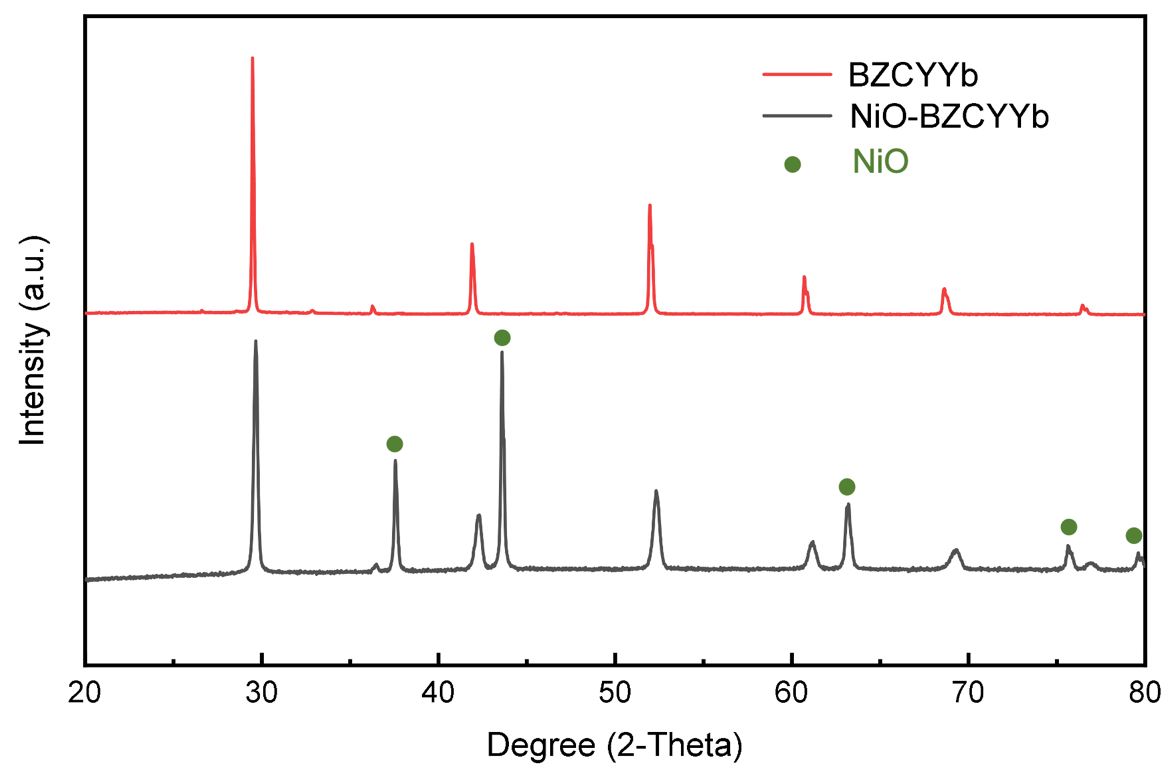


**Fig. S20** XRD patterns of a dense BZCYYb4411 electrolyte and NiO-BZCYYb4411 composite cathode


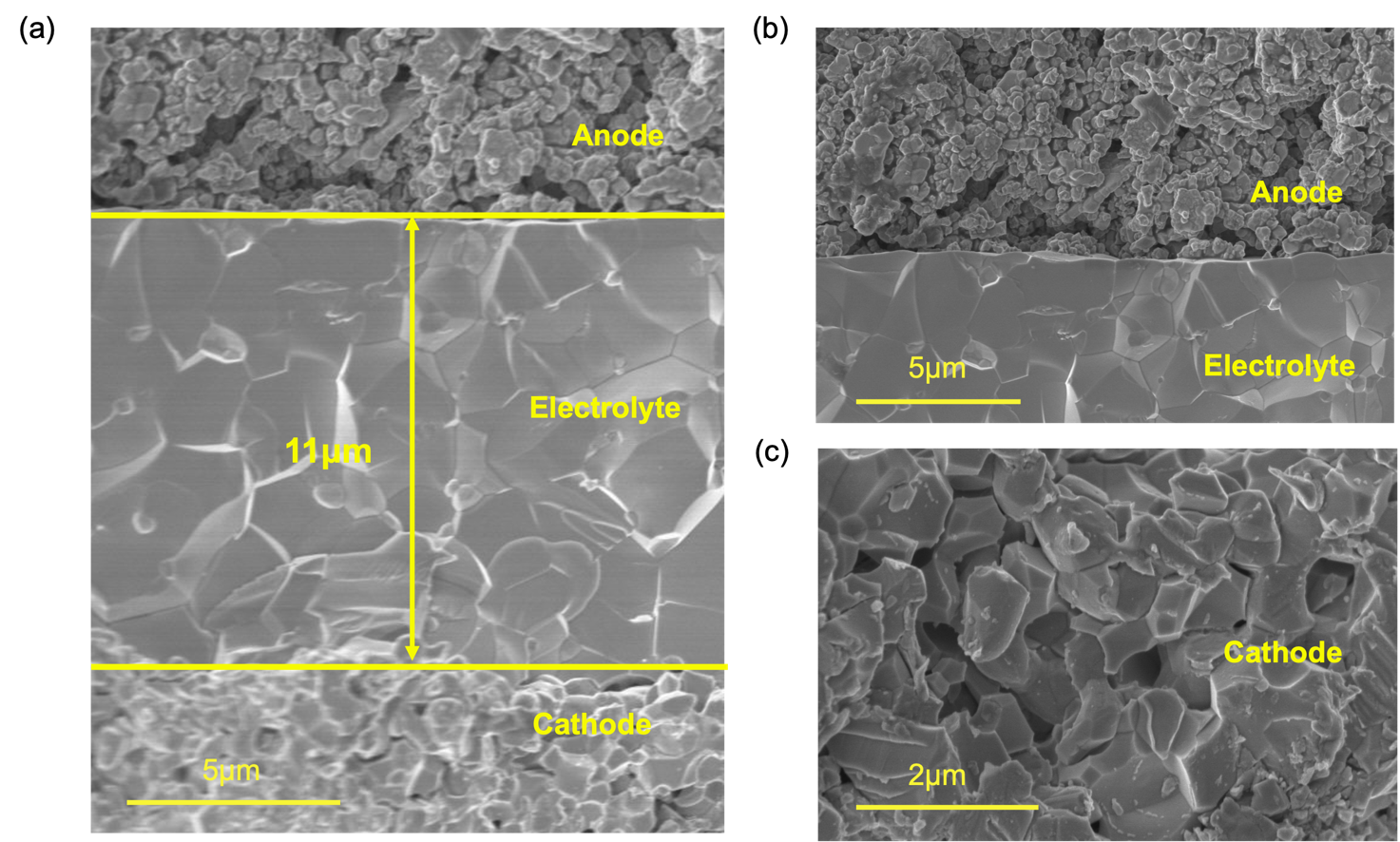


**Fig. S21** SEM images of P-SOEC with LBCN9173 anode: (**a**) cross-section, (**b**) magnified image near the interface between anode and electrolyte, and (**c**) cathode


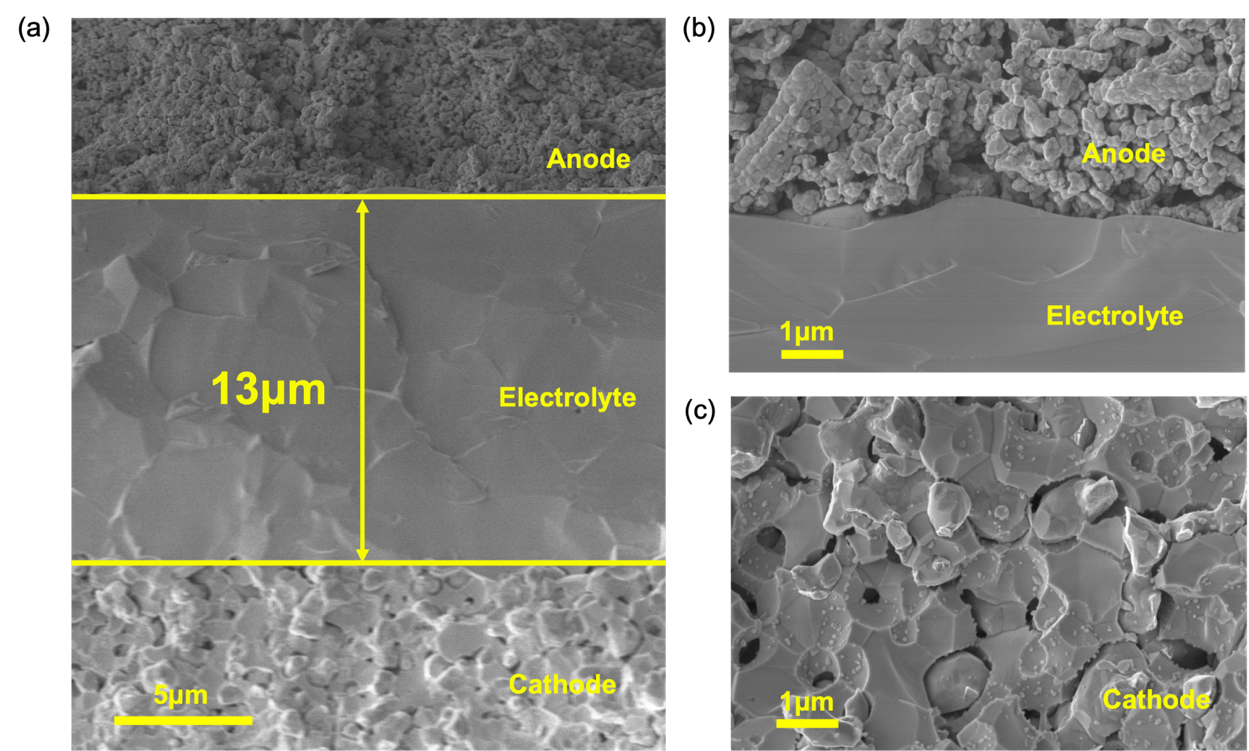


**Fig. S22** SEM images of P-SOECs with LCCN9173 as the anode: (**a**) cross-section, (**b**) magnified image near the interface between anode and electrolyte, and (**c**) cathode


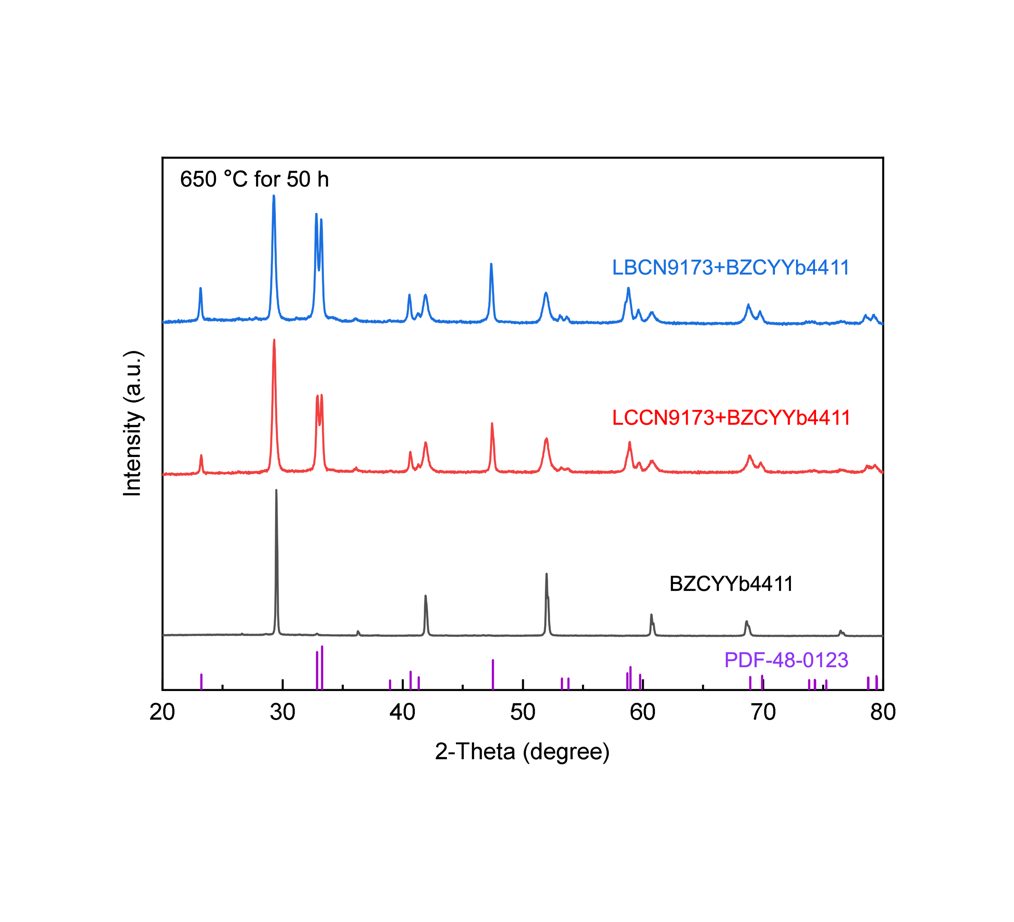


**Fig. S23** XRD patterns of LBCN9173/LCCN9173+BZCYYb4411 composite oxides (with a mass ratio 1:1) after exposing to air for 50 h at 650 °C and raw BZCYYb4411 oxide


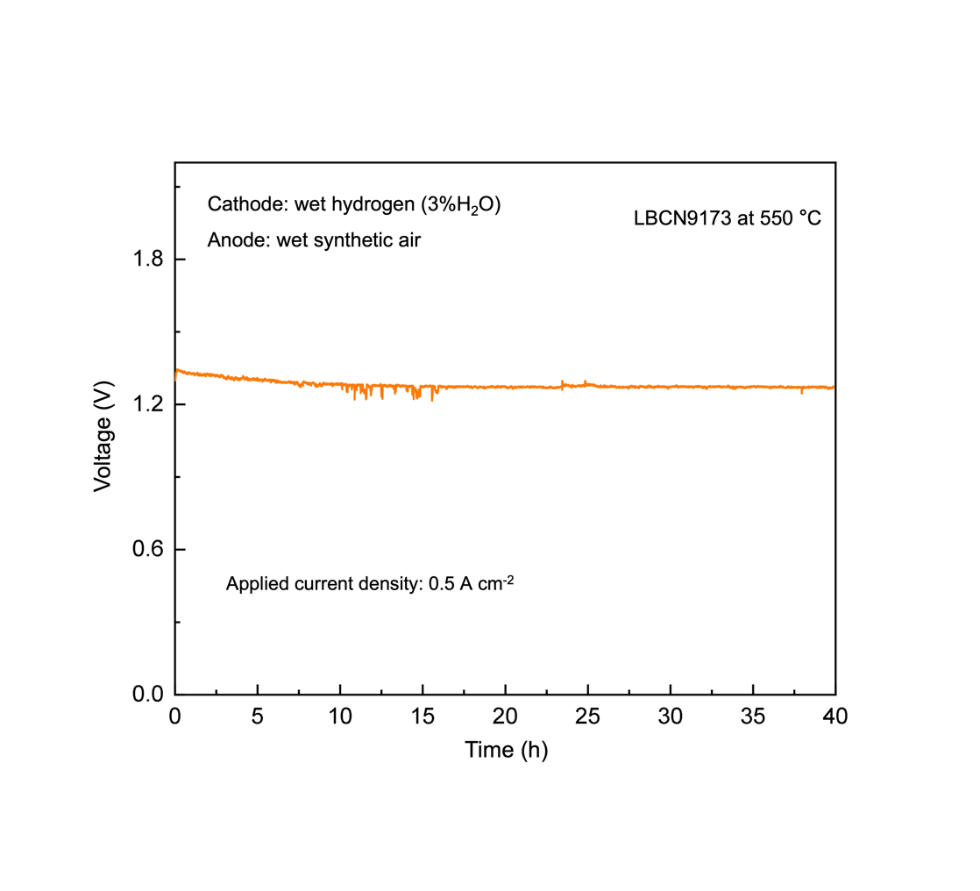


**Fig. S24** The stability testing of P-SOEC with LBCN9173 anode under wet synthetic air at a current density of 0.5 A cm^-2^ and 550 °C


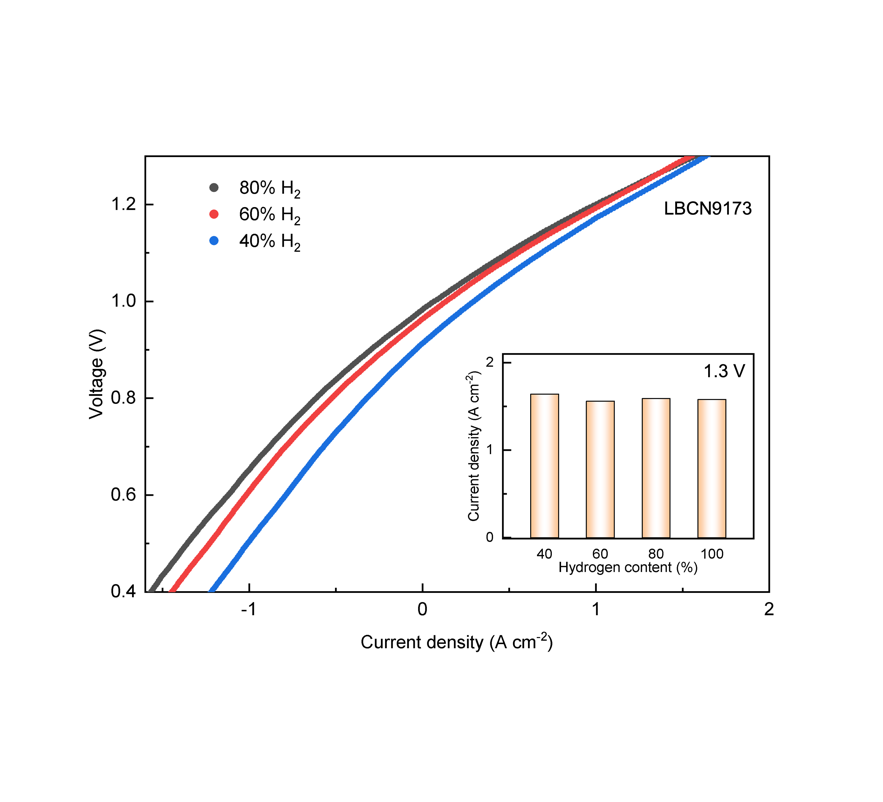


**Fig. S25** I-V curves of P-SOEC with LBCN9173 anode under 40%, 60%, and 80% hydrogen content, respectively, at 600 °C


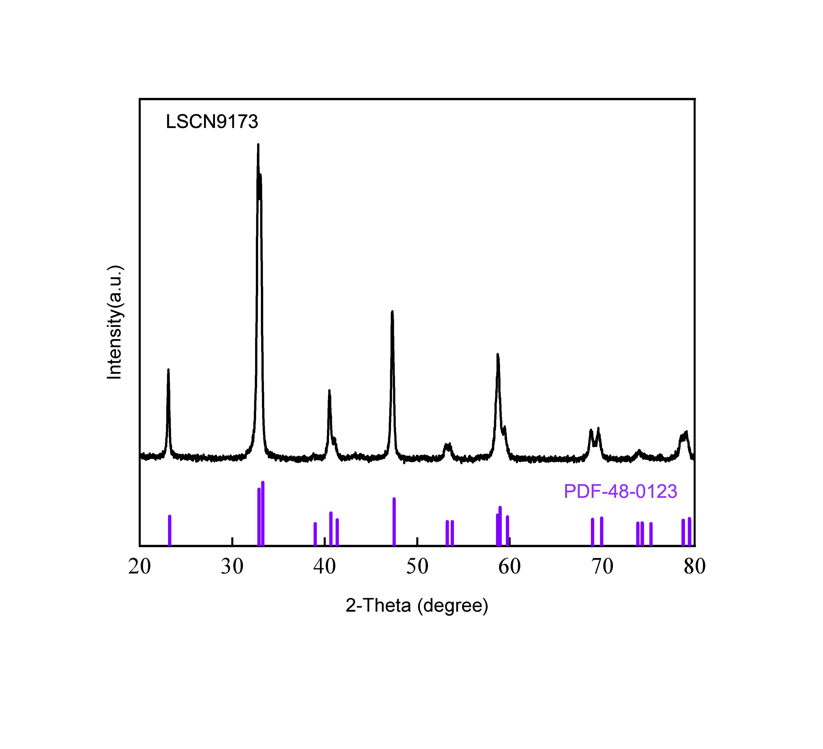


**Fig. S26** XRD pattern of LSCN9173 oxide


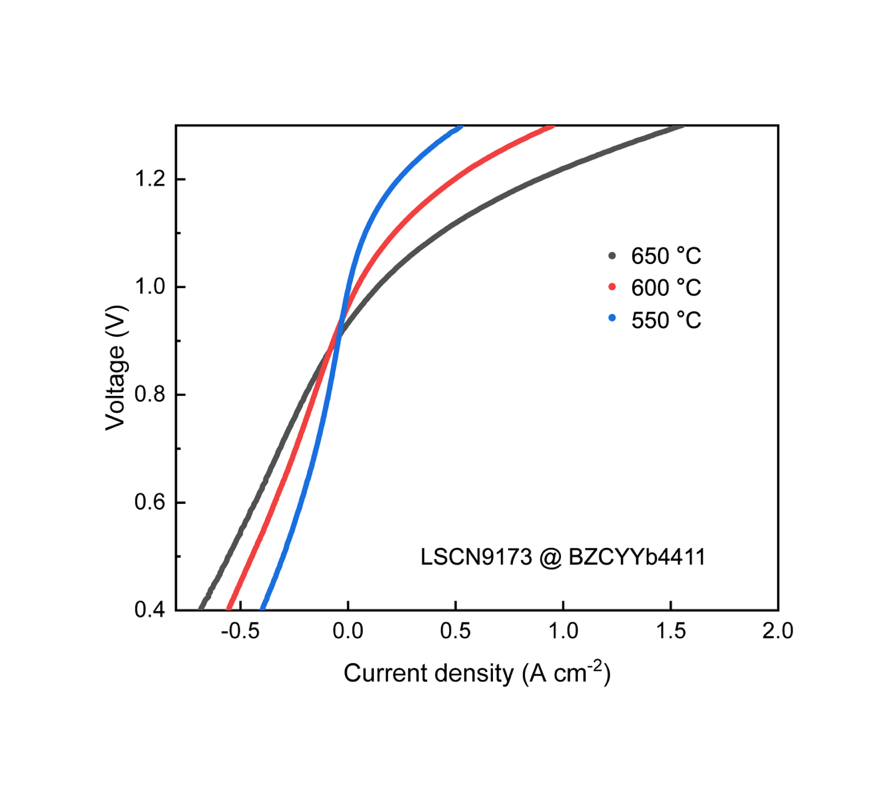


**Fig. S27** I-V curves of P-SOEC with LSCN9173 anode at 550-650 °C


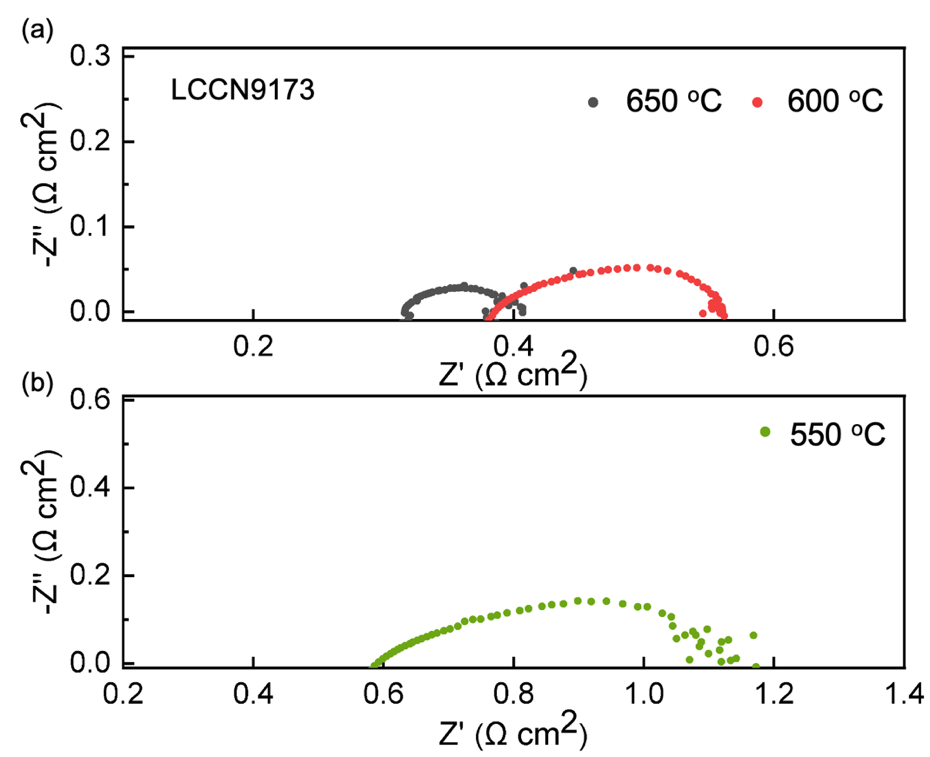


**Fig. S28** EIS of P-SOEC with LCCN9173 anode measured at (**a**) 650 °C, 600 °C and (**b**) 550 °C


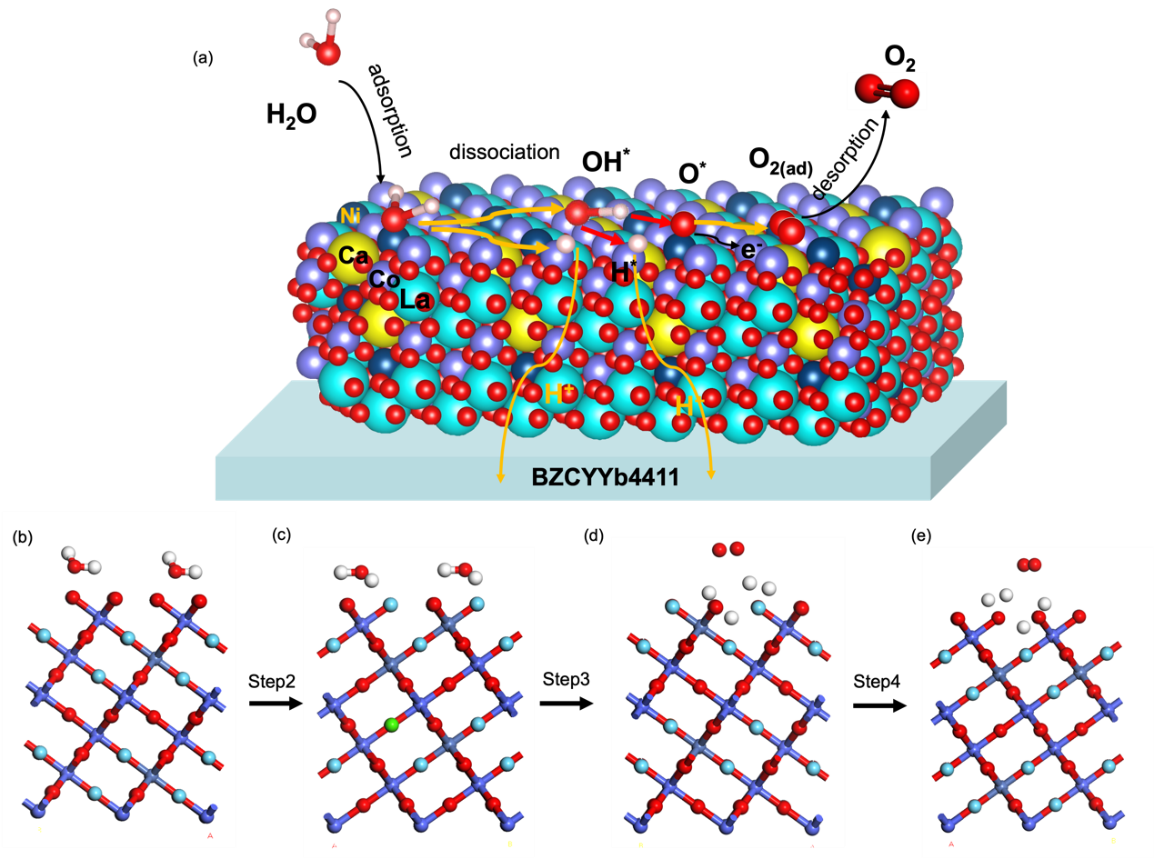


**Fig. S29** Schematic WOR reaction mechanism on (001) surface of LCCN9173 oxide


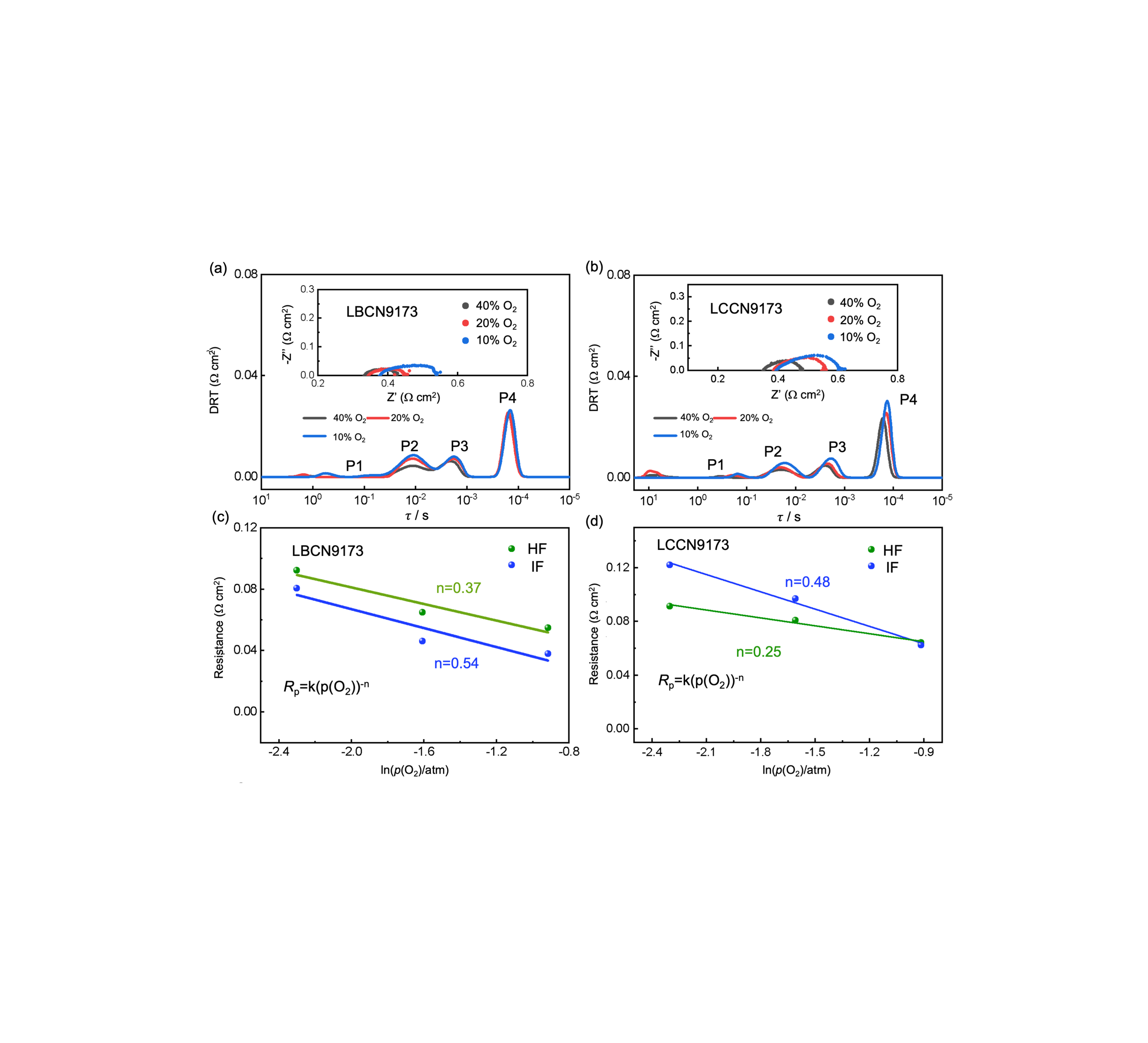


**Fig. S30** DRT plots and EIS of (**a**) LBCN9173 and (**b**) LCCN9173 cell as a function of *p*_O2_ (*p*_O2_=10%, 20%, 40%) at 600 °C. Plots of R_HF_ and R_IF_ dependences *p*_O2_ for (**c**) LBCN9173 and (**d**) LCCN9173 cell


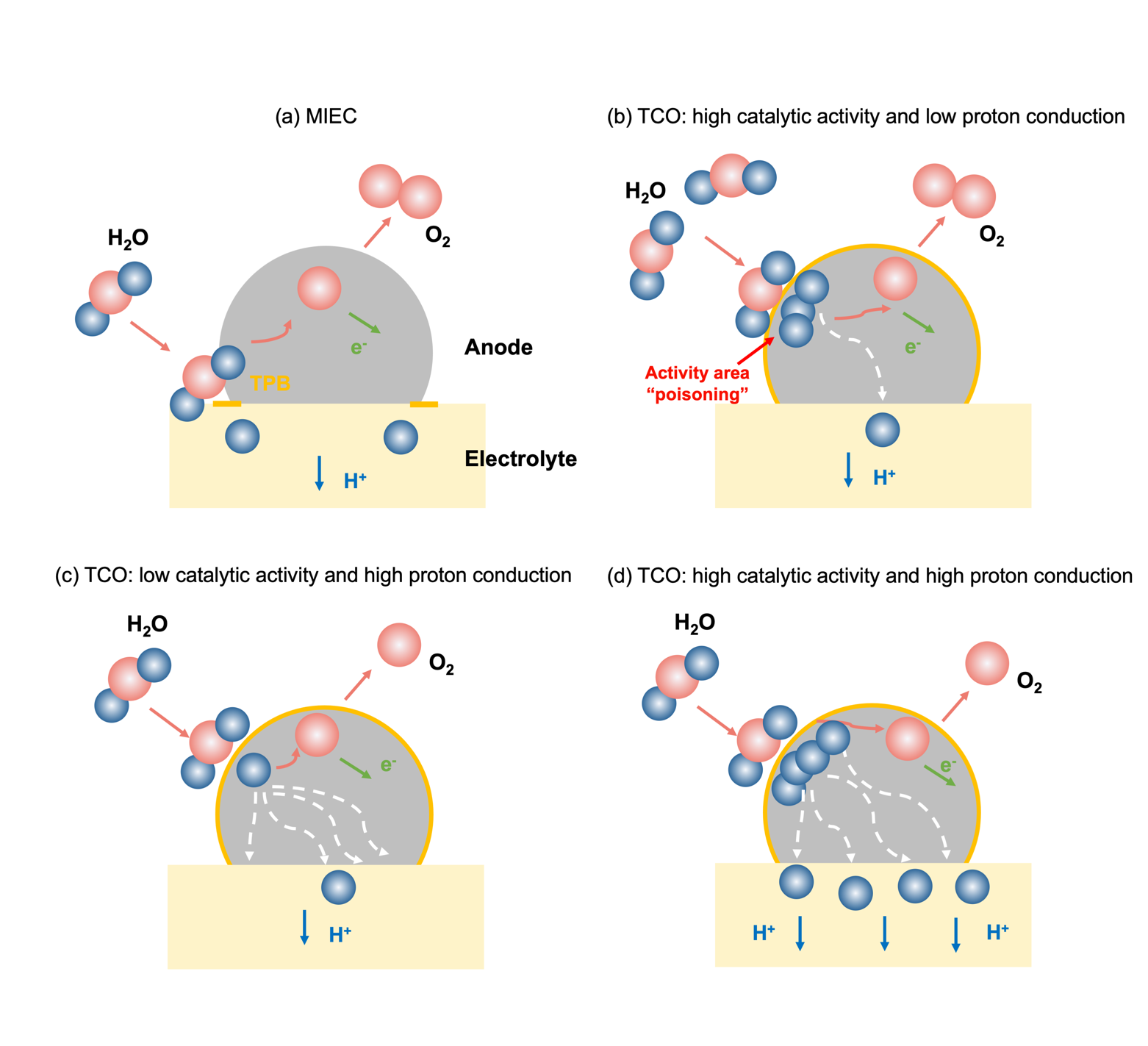


**Fig. S31** Schematic diagram of the anodic reaction

**Table S1** XRD Refinement results of LBCN9173 and LCCN9173 oxides

| Composition | a(Å) | b(Å) | c(Å) | R_p_ (%) | R_wp_ (%) | R_exp_ (%) | Space group |
| --- | --- | --- | --- | --- | --- | --- | --- |
| LBCN9173 | 5.4547 | 5.4547 | 13.13578 | 2.76 | 3.66 | 0.85 | $\text{R}\bar{\text{3}}\text{c}$ |
| LCCN9173 | 5.4476 | 5.4476 | 13.12905 | 3.87 | 4.88 | 1.01 | $\text{R}\bar{\text{3}}\text{c}$ |

**Table S2** XPS of O 1s spectra fitting results for dehydrated and hydrated LBCN9173 and LCCN9173 samples

| Samples | | Dehydrated  LBCN9173 | | Hydrated  LBCN9173 | | Dehydrated  LCCN9173 | | Hydrated  LCCN9173 | |
| --- | --- | --- | --- | --- | --- | --- | --- | --- | --- |
|  |  | Position (eV) | Area percentage (%) | Position (eV) | Area percentage (%) | Position (eV) | Area percentage (%) | Position (eV) | Area percentage (%) |
| O1s | O^2-^ | 528.70 | 36.33  9.85  44.04  9.78 | 528.38 | 35.87  7.56  47.60  8.96 | 528.33 | 37.47  8.25  46.17  8.11 | 528.33 | 39.82  7.14  47.01  6.02 |
|  | $\text{V}_{\text{O}}^{\text{••}}$ | 529.66 |  | 529.40 |  | 529.38 |  | 529.38 |  |
|  | O_OH_ | 531.09 |  | 530.98 |  | 530.90 |  | 530.93 |  |
|  | O_H2O_ | 532.85 |  | 532.90 |  | 532.99 |  | 532.98 |  |

**Table S3** Energies for dehydrated and hydrated LBCN9173 and LCCN9173 oxides with oxygen vacancy at different sites

| LBCN9173 | E_dehyd_ (eV) | E_perfect_  (eV) | 1/2E_O2_  (eV) | ΔE_FE_  (eV) | E_hyd_  (eV) | E_H2O_  (eV) | ΔE_hydration_  (eV) |
| --- | --- | --- | --- | --- | --- | --- | --- |
| La-$\text{V}_{\text{O}}^{\text{••}}$-La | -250.91 | -253.30 | -0.1668 | 2.23 | -266.04 | -13.49 | -1.64 |
| La-$\text{V}_{\text{O}}^{\text{••}}$-Ba | -251.16 | -253.30 | -0.1568 | 1.99 | -266.72 | -13.49 | -2.06 |
| Ba-$\text{V}_{\text{O}}^{\text{••}}$-Ba | -251.47 | -253.30 | -0.1568 | 1.67 | -267.27 | -13.49 | -2.30 |
| LCCN9173 | E_dehyd_ (eV) | E_perfect_ (eV) | 1/2E_O2_ (eV) | ΔE_FE_ (eV) | E_hyd_  (eV) | E_H2O_  (eV) | ΔE_hydration_  (eV) |
| La-$\text{V}_{\text{O}}^{\text{••}}$-La | -841.49 | -844.30 | -0.168 | 2.64 | -855.05 | -13.49 | -0.06 |
| La-$\text{V}_{\text{O}}^{\text{••}}$-Ca | -841.62 | -844.30 | -0.168 | 2.52 | -855.33 | -13.49 | -0.22 |
| Ca-$\text{V}_{\text{O}}^{\text{••}}$-Ca | -842.01 | -844.30 | -0.187 | 2.10 | -856.52 | -13.49 | -1.02 |

**Table S4** Proton conducting energy barrier values at different steps

| O1-O2/steps | LBCN9173 | LCCN9173 | O2-O3/steps | LBCN9173 | LCCN9173 |
| --- | --- | --- | --- | --- | --- |
| 1 | 0.14 | 0.19 | 1 | 0.11 | 0.21 |
| 2 | 0.02 | 0.12 | 2 | 0.06 | 0.12 |
| 3 | 0.22 | 0.35 | 3 | 0.38 | 0.48 |
| 4 | 0.43 | 0.57 | 4 | 0.74 | 0.79 |
| 5 | 0.18 | 0.43 | 5 | 0.48 | 0.47 |

**Table S5** TEC values of this work, Co-base and Co-free oxides

| Anode composition | TEC (10^-6^ K^-1^) | Refs |
| --- | --- | --- |
| La_0.6_Sr_0.4_Co_0.2_Fe_0.8_O_3-_*_δ_* | 21.4 | [S4] |
| Ba_0.5_Sr_0.5_Co_0.8_Fe_0.2_O_3-_*_δ_* | 19.7 | [S5] |
| BaCo_0.4_Fe_0.4_Zr_0.1_Y_0.1_O_3-δ_ | 20.4 | [S6] |
| PrNi_0.5_Co_0.5_O_3-_*_δ_* | 19.2 | [S7] |
| La_0.6_Sr_0.4_Fe_0.8_Ni_0.2_O_3-_*_δ_* | 13.7 | [S8] |
| Pr_0.5_Sr_0.5_Fe_0.8_Cu_0.2_O_3-_*_δ_* | 16.4 | [S9] |
| La_0.4_Ca_0.6_Fe_0.8_Cr_0.2_O_3-_*_δ_* | 13.2 | [S10] |
| La_0.9_Ca_0.1_Co_0.7_Ni_0.3_O_3-δ_ | 15.1 | This work |
| La_0.9_Ba_0.1_Co_0.7_Ni_0.3_O_3-δ_ | 15.4 | This work |

**Table S6** Comparison of current densities at 600 °C and 1.3V for P-SOECs with high-performance anodes

| Time | Materials | | Inlet gas composition | | *I*  (A cm^–2^) |
| --- | --- | --- | --- | --- | --- |
|  | Anode | Electrolyte | Anode | Fuel electrode |  |
| 2020[**S**11] | PNC55 (3D) | BZCYYb4411 | Air (10% H_2_O) | 10% H_2_/Ar | 1.17 |
| 2020**[**S12] | PBCC95 | BZCYYb4411 | Air (20% H_2_O) | 100% H_2_(3% H_2_O) | 0.72 |
| 2021[S13] | SCFN | BZCYYb1711 | Air | 100% H_2_ | 0.36 |
| 2022[S14] | BCFZYM | BZCYYb1711 | Air (10% H_2_O) | 100% H_2_(3% H_2_O) | 1.24 |
| 2022[S15] | BSCFP | BZCYYb1711 | Air (10% H_2_O) | 100% H_2_ | 1 |
| 2022[S16] | PBCFN | BZCYYb1711 | Air (3% H_2_O) | 100% H_2_(3% H_2_O) | 1.04 |
| 2022[S17] | LCCN7382 | BZCYYb4411 | Air (3% H_2_O) | 100% H_2_(3% H_2_O) | 0.88 |
| 2023[S18] | LCN91 | BZCYYb4411 | Air (3% H_2_O) | 100% H_2_(3% H_2_O) | 0.84 |
| 2023[S19] | BCFNbS | BZCYYb1711 | Air (3% H_2_O) | 100% H_2_(3% H_2_O) | 0.91 |
| 2023[S20] | BSCFE | BZCYYb1711 | Air (5% H_2_O) | 100% H_2_(3% H_2_O) | 0.98 |
| 2023[S21] | BCFZYN | BZCYYb1711 | Air (5% H_2_O) | 100% H_2_(3% H_2_O) | 1.2 |
| 2024[S22] | SFN | BZCYYb1711 | Air (3% H_2_O) | 100% H_2_ | 0.8 |
| 2024[S23] | PSCN | BZCYYb1711 | Air | 100% H_2_(3% H_2_O) | 1.25 |
| 2024[S24] | BCFZYNF | BZCYYb1711 | Air (3% H_2_O) | 100% H_2_ | 1.1 |
| 2025[S25] | BSCsCFZr | BZCYYb1711 | Air (3% H_2_O) | 97% H_2_(3% H_2_O) | 1.23 |
| 2025[S26] | LCCFN‐Cr | BZCYYb6211 | Air (3% H_2_O) | 97% H_2_(3% H_2_O) | 1.18 |
| 2025[S27] | BLFZN0.1 | BZCYYb1711 | Air (3% H_2_O) | 97% H_2_(3% H_2_O) | 0.67 |
| This work | LSCN9173 | BZCYYb4411 | Air (3% H_2_O) | 97% H_2_(3% H_2_O) | 0.95 |
| This work | LCCN9173 | BZCYYb4411 | Air (3% H_2_O) | 97% H_2_(3% H_2_O) | 1.01 |
| This work | LBCN9173 | BZCYYb4411 | Air (3% H_2_O) | 97% H_2_(3% H_2_O) | 1.58 |

PNC=PrNi_0.5_Co_0.5_O_3-δ_ [S11], PBCC95=(PrBa_0.8_Ca_0.2_)_0.95_Co_2_O_6−δ_ [S12], SCFN= Sr_0.9_Ce_0.1_Fe_0.8_Ni_0.2_O_3-δ_ [S13], BCFZYM=Ba(Co_0.4_Fe_0.4_Zr_0.1_Y_0.1_)_0.95_Mg_0.05_O_3-δ_ [S11], BSCFP= Ba_0.5_Sr_0.5_(Co_0.8_Fe_0.2_)_0.95_P_0.05_O_3-δ_ [S15], PBCFN=PrBaCo_1.6_Fe_0.2_Nb_0.2_O_5+δ_ [S16], LCCN7382=La_0.7_Ca_0.3_Co_0.8_Ni_0.2_O_3-δ_[S17], LCN91= LaCo_0.9_Ni_0.1_O_3-δ_ [S18], BCFNS= BaCo_0.4_Fe_0.4_Nb_0.1_Sc_0.1_O_3-δ_ [S19], BSCFE= Ba_0.5_Sr_0.5_(Co_0.8_Fe_0.2_)_0.9_Er_0.1_O_3-δ_ [S20], BCFZYN= Ba_0.95_(Co_0.4_Fe_0.4_Zr_0.1_Y_0.1_)_0.95_Ni_0.05_O_3-δ_ [S21], SFN= Sr_2.8_Fe_1.8_Nb_0.2_O_7-δ_ [S22], PSCN= PrSrCo_1.8_Nb_0.2_O_6-δ_ [S23], BCFZYNF= Ba(Co_0.4_Fe_0.4_Zr_0.1_Y_0.1_)_0.95_Ni_0.05_F_0.1_O_2.9-δ_ [S24], BSCsCFZr= Ba_0.4_Sr_0.5_Cs_0.1_Co_0.7_Fe_0.2_Zr_0.1_O_3−δ_ [S25], BLFZN0.1= Ba_0.95_La_0.05_(Fe_0.8_Zn_0.2_)_0.9_Ni_0.1_O_3-δ_ [S27], LCCFN‐Cr= La(Co_0.2_Cu_0.2_Fe_0.2_Ni_0.2_Cr_0.2_)O_3-δ_ [S26], BZCYYb1711= BaZr_0.1_Ce_0.7_Y_0.1_Yb_0.1_O_3-δ_.

**Table S7** R_HF,_ R_IF_, R_LF_ values and ratios for LBCN9173 and LCCN9173 at 550 and 600 °C

| Reaction steps | R (Peaks) | Reaction equations | Resistance from DRT peak (Ω cm^2^) | | | | |
| --- | --- | --- | --- | --- | --- | --- | --- |
|  |  |  | 550 °C | | 600 °C | 550 °C | 600 °C |
| step1: | R_IF_  (P2-P3) | $\text{2H}_{\text{2}}\text{O }\text{⟶}\text{ }{\text{2H}_{\text{2}}\text{O}}^{\text{*}}$  (H_2_O adsorption) | LCCN9173 | | | LBCN9173 | |
| step2: |  | ${\text{2H}_{\text{2}}\text{O}}^{\text{*}}\text{ }\text{⟶}\text{ }\text{2OH}^{\text{*}}\text{+2}\text{H}^{\text{*}}$  (H_2_O^*^ dissociation) | 0.364 | 0.094 | | 0.213 | 0.039 |
| step3: |  | $\text{2OH}^{\text{*}}\text{⟶}\text{ 2}\text{O}^{\text{*}}\text{+}\text{2H}^{\text{*}}$  (OH^*^ dissociation) |  |  |  |  |  |
| step4: |  | $\text{2O}^{\text{*}}\text{- 4}\text{e}^{\text{-}}\text{⟶}{\text{ O}_{\text{2}}}^{\text{*}}$  (O_2_ formation) |  |  |  |  |  |
| step5: | R_LF_  (P1) | ${\text{O}_{\text{2}}}^{\text{*}}\text{ }\text{⟶}\text{ }\text{O}_{\text{2}}$  (O_2_ desorption) | 0.028 | 0.003 | | 0.022 | 0.007 |
| step6: | R_HF_  (P4) | $\text{OH}_{\text{O anode}}^{\text{•}}\text{⟶}\text{ }\text{OH}_{\text{O electrolyte}}^{\text{•}}$  (Charge transfer) | 0.148 | 0.078 | | 0.158 | 0.055 |

# Supplementary References

1. W. Zhou, Z. Shao, R. Ran, P. Zeng, H. Gu et al., Ba_0.5_Sr_0.5_Co_0.8_Fe_0.2_O_3−δ_+LaCoO_3_ composite cathode for Sm_0.2_Ce_0.8_O_1.9_-electrolyte based intermediate-temperature solid-oxide fuel cells. J. Power Sources **168**, 330-337 (2007). <https://doi.org/10.1016/j.jpowsour.2007.03.041>
2. A.N. Petrov, O.F. Kononchuk, A.V. Andreev, V.A. Cherepanov, P. Kofstad, Crystal structure, electrical and magnetic properties of La_1-x_Sr_x_CoO_3-y_. Solid State Ionics **80**, 189-199 (1995). <https://doi.org/10.1016/0167-2738(95)00114-L>
3. R. Kun, S. Populoh, L. Karvonen, J. Gumbert, A. Weidenkaff et al., Structural and thermoelectric characterization of Ba substituted LaCoO_3_ perovskite-type materials obtained by polymerized gel combustion method. J. Alloys Compd. **579**, 147-155 (2013). <https://doi.org/10.1016/j.jallcom.2013.05.019>
4. G.C. Kostogloudis, C. Ftikos, Properties of A-site-deficient La_0.6_Sr_0.4_Co_0.2_Fe_0.8_O_3−δ_-based perovskite oxides. Solid State Ionics **126**, 143-151 (1999). <https://doi.org/10.1016/S0167-2738(99)00230-1>
5. S. Li, Z. Lü, B. Wei, X. Huang, J. Miao et al., A study of (Ba_0.5_Sr_0.5_)_1−x_Sm_x_Co_0.8_Fe_0.2_O_3−δ_ as a cathode material for IT-SOFCs. J. Alloys Compd. **426**, 408-414 (2006). <https://doi.org/10.1016/j.jallcom.2006.02.040>
6. M. Liang, F. He, C. Zhou, Y. Chen, R. Ran et al., Nickel-doped BaCo_0.4_Fe_0.4_Zr_0.1_Y_0.1_O_3-δ_ as a new high-performance cathode for both oxygen-ion and proton conducting fuel cells. Chem. Eng. J. **420**, 127717 (2021). <https://doi.org/10.1016/j.cej.2020.127717>
7. Z.Y. Zhu, M.Y. Zhou, K. Tan, Z.D. Fan, D. Cao et al., High performance and stability enabled by tuning the component thermal expansion coefficients of a proton-conducting solid oxide cell operating at high steam concentration. ACS Appl. Mater. Interfaces **15**, 14457-14469 (2023). <https://doi.org/10.1021/acsami.3c00728>
8. S.P. Simner, J.F. Bonnett, N.L. Canfield, K.D. Meinhardt, V.L. Sprenkle et al., Optimized lanthanum ferrite-based cathodes for anode-supported SOFCs. Electrochem. Solid St **5**, A173-A175 (2002). <https://doi.org/10.1149/1.1483156>
9. S. Pang, W. Wang, T. Chen, X. Shen, Y. Wang et al., Systematic evaluation of cobalt-free Ln_0.5_Sr_0.5_Fe_0.8_Cu_0.2_O_3−δ_ (Ln = La, Pr, and Nd) as cathode materials for intermediate-temperature solid oxide fuel cells. J. Power Sources **326**, 176-181 (2016). <https://doi.org/10.1016/j.jpowsour.2016.06.134>
10. J. Xiao, Q. Xu, M. Chen, K. Zhao, B.H. Kim, Improved overall properties in La_1-x_Ca_x_Fe_0.8_Cr_0.2_O_3-δ_ as cathode for intermediate temperature solid oxide fuel cells. Ionics **21**, 2805-2814 (2015). <https://doi.org/10.1007/s11581-015-1468-1>
11. H. Ding, W. Wu, C. Jiang, Y. Ding, W. Bian et al., Self-sustainable protonic ceramic electrochemical cells using a triple conducting electrode for hydrogen and power production. Nat. Commun. **11**, 1907 (2020). <https://doi.org/10.1038/s41467-020-15677-z>
12. W. Tang, H. Ding, W. Bian, W. Wu, W. Li et al., Understanding of A-site deficiency in layered perovskites: promotion of dual reaction kinetics for water oxidation and oxygen reduction in protonic ceramic electrochemical cells. J. Mater. Chem. A **8**, 14600-14608 (2020). <https://doi.org/10.1039/d0ta05137c>
13. Y. Song, J. Liu, Y. Wang, D. Guan, A. Seong et al., Nanocomposites: a new opportunity for developing highly active and durable bifunctional air electrodes for reversible protonic ceramic cells. Adv. Energy Mater. **11**, 2101899 (2021). <https://doi.org/10.1002/aenm.202101899>
14. M. Liang, Y. Song, D. Liu, L. Xu, M. Xu et al., Magnesium tuned triple conductivity and bifunctionality of BaCo_0.4_Fe_0.4_Zr_0.1_Y_0.1_O_3-δ_ perovskite towards reversible protonic ceramic electrochemical cells. Appl. Catal. B Environ. **318**, 121868 (2022). <https://doi.org/10.1016/j.apcatb.2022.121868>
15. Z. Liu, D. Cheng, Y. Zhu, M. Liang, M. Yang et al., Robust bifunctional phosphorus-doped perovskite oxygen electrode for reversible proton ceramic electrochemical cells. Chem. Eng. J. **450**, 137787 (2022). <https://doi.org/10.1016/j.cej.2022.137787>
16. K. Xu, H. Zhang, Y. Xu, F. He, Y. Zhou et al., An efficient steam‐induced heterostructured air electrode for protonic ceramic electrochemical cells. Adv. Funct. Mater. **32**, 2110998 (2022). <https://doi.org/10.1002/adfm.202110998>
17. N. Wang, B. Yuan, C. Tang, L. Du, R. Zhu et al., Machine‐learning‐accelerated development of efficient mixed protonic–electronic conducting oxides as the air electrodes for protonic ceramic cells. Adv. Mater. **34**, 2203446 (2022). <https://doi.org/10.1002/adma.202203446>
18. N. Wang, B. Yuan, F. Zheng, S. Mo, X. Zhang et al., Machine‐learning assisted screening proton conducting Co/Fe based oxide for the air electrode of protonic solid oxide cell. Adv. Funct. Mater. **34**, 2309855 (2023). <https://doi.org/10.1002/adfm.202309855>
19. C. Lu, R. Ren, Z. Zhu, G. Pan, G. Wang et al., BaCo_0.4_Fe_0.4_Nb_0.1_Sc_0.1_O_3-δ_ perovskite oxide with super hydration capacity for a high-activity proton ceramic electrolytic cell oxygen electrode. Chem. Eng. J. **472**, 144878 (2023). <https://doi.org/10.1016/j.cej.2023.144878>
20. Z. Liu, Y. Lin, H. Nie, D. Liu, Y. Li et al., Highly active nanocomposite air electrode with fast proton diffusion channels via Er doping‐induced phase separation for reversible proton ceramic electrochemical cells. Adv. Funct. Mater. **34**, 2311140 (2024). <https://doi.org/10.1002/adfm.202311140>
21. M. Liang, Y. Wang, Y. Song, D. Guan, J. Wu et al., High-temperature water oxidation activity of a perovskite-based nanocomposite towards application as air electrode in reversible protonic ceramic cells. Appl. Catal. B Environ. **331**, 122682 (2023). <https://doi.org/10.1016/j.apcatb.2023.122682>
22. N. Yu, I.T. Bello, X. Chen, T. Liu, Z. Li et al., Rational design of ruddlesden–popper perovskite ferrites as air electrode for highly active and durable reversible protonic ceramic cells. Nano-Micro Lett. **16**, 177 (2024). <https://doi.org/10.1007/s40820-024-01397-2>
23. L.Z. Kang Zhu, N. Shi, B. Qiu, X. Hu, D. Huan et al., A superior catalytic air electrode with temperature-induced exsolution toward protonic ceramic cells. ACS Nano **18**, 5141-5151 (2024). <https://doi.org/10.1021/acsnano.3c12609>
24. X. Chen, N. Yu, Y. Song, T. Liu, H. Xu et al., Synergistic bulk and surface engineering for expeditious and durable reversible protonic ceramic electrochemical cells air electrode. Adv. Mater. **36**, 2403998 (2024). <https://doi.org/10.1002/adma.202403998>
25. Y. Zhang, Y. Wang, Z. Liu, Z. Wang, Y. Wang et al., Constructing robust and efficient ceramic cells air electrodes through collaborative optimization bulk and surface phases. Adv. Funct. Mater. 2422531 (2025). <https://doi.org/10.1002/adfm.202422531>
26. X. Zhang, C. Tang, Y. Yang, F. Zheng, Q. Su et al., Novel high-entropy air electrodes enhancing electrochemical performances of reversible protonic ceramic cells. Adv. Funct. Mater. 2421083 (2025). <https://doi.org/10.1002/adfm.202421083>
27. X. Yu, L. Ge, Y. Mi, B. Wu, Z. Yu et al., Superior active and durable air electrode for protonic ceramic cells by metal-oxide bond engineering. Small **21**, 2408607 (2025). <https://doi.org/10.1002/smll.202408607>
